# A low-gluten diet induces changes in the intestinal microbiome of healthy Danish adults

**DOI:** 10.1038/s41467-018-07019-x

**Published:** 2018-11-13

**Authors:** Lea B. S. Hansen, Henrik M. Roager, Nadja B. Søndertoft, Rikke J. Gøbel, Mette Kristensen, Mireia Vallès-Colomer, Sara Vieira-Silva, Sabine Ibrügger, Mads V. Lind, Rasmus B. Mærkedahl, Martin I. Bahl, Mia L. Madsen, Jesper Havelund, Gwen Falony, Inge Tetens, Trine Nielsen, Kristine H. Allin, Henrik L. Frandsen, Bolette Hartmann, Jens Juul Holst, Morten H. Sparholt, Jesper Holck, Andreas Blennow, Janne Marie Moll, Anne S. Meyer, Camilla Hoppe, Jørgen H. Poulsen, Vera Carvalho, Domenico Sagnelli, Marlene D. Dalgaard, Anders F. Christensen, Magnus Christian Lydolph, Alastair B. Ross, Silas Villas-Bôas, Susanne Brix, Thomas Sicheritz-Pontén, Karsten Buschard, Allan Linneberg, Jüri J. Rumessen, Claus T. Ekstrøm, Christian Ritz, Karsten Kristiansen, H. Bjørn Nielsen, Henrik Vestergaard, Nils J. Færgeman, Jeroen Raes, Hanne Frøkiær, Torben Hansen, Lotte Lauritzen, Ramneek Gupta, Tine Rask Licht, Oluf Pedersen

**Affiliations:** 1 Department of Bio and Health Informatics, Technical University of Denmark, DK-2800 Kgs. Lyngby, Denmark; 2 National Food Institute, Technical University of Denmark, DK-2800 Kgs. Lyngby, Denmark; 3 Department of Nutrition, Exercise and Sports, Faculty of Science, University of Copenhagen, DK-1958 Frederiksberg, Denmark; 4 The Novo Nordisk Foundation Center for Basic Metabolic Research, University of Copenhagen, DK-2200 Copenhagen, Denmark; 5 Department of Microbiology and Immunology, KU Leuven–University of Leuven, Rega Institute, 3000 Leuven, Belgium; 6 VIB, Center for Microbiology, 3000 Leuven, Belgium; 7 Department of Veterinary Disease Biology, Faculty of Science, University of Copenhagen, DK-1958 Frederiksberg, Denmark; 8 Department of Biochemistry and Molecular Biology, University of Southern Denmark, DK-5230 Odense, Denmark; 9 Department of Biomedical Sciences, University of Copenhagen, Copenhagen, DK-2200 Denmark; 10 Department of Radiology, Bispebjerg Hospital, Copenhagen, DK-2400 Denmark; 11 Department of Chemical and Biochemical Engineering, Technical University of Denmark, DK-2800 Kgs. Lyngby, Denmark; 12 Department of Plant and Environmental Sciences, University of Copenhagen, DK-1958 Frederiksberg, Denmark; 13 Department of Biotechnology and Biomedicine, Technical University of Denmark, DK-2800 Kgs. Lyngby, Denmark; 14 Department of Clinical Biochemistry, Copenhagen University Hospital Hvidovre, DK-2650 Hvidovre, Denmark; 15 Department of Autoimmunology & Biomarkers, Statens Serum Institut, DK-2300 Copenhagen, Denmark; 16 Department of Biology and Biological Engineering, Chalmers University of Technology, 412 96 Gothenburg, Sweden; 17 School of Biological Sciences, The University of Auckland, 1010 Auckland, New Zealand; 18 Bartholin Institute, Rigshospitalet, DK-2200 Copenhagen, Denmark; 19 Research Centre for Prevention and Health, The Capital Region of Denmark, DK-2000 Frederiksberg, Denmark; 20 Research Unit and Department of Gastroenterology, Herlev and Gentofte Hospital, the Capital Region of Denmark, 2730 Herlev, Denmark; 21 Biostatistics, Department of Public Health, University of Copenhagen, DK-1014 Copenhagen, Denmark; 22 Laboratory of Genomics and Molecular Biomedicine, Department of Biology, University of Copenhagen, DK-2100 Copenhagen, Denmark; 23 Clinical-Microbiomics A/S, DK-2200 Copenhagen, Denmark

## Abstract

Adherence to a low-gluten diet has become increasingly common in parts of the general population. However, the effects of reducing gluten-rich food items including wheat, barley and rye cereals in healthy adults are unclear. Here, we undertook a randomised, controlled, cross-over trial involving 60 middle-aged Danish adults without known disorders with two 8-week interventions comparing a low-gluten diet (2 g gluten per day) and a high-gluten diet (18 g gluten per day), separated by a washout period of at least six weeks with habitual diet (12 g gluten per day). We find that, in comparison with a high-gluten diet, a low-gluten diet induces moderate changes in the intestinal microbiome, reduces fasting and postprandial hydrogen exhalation, and leads to improvements in self-reported bloating. These observations suggest that most of the effects of a low-gluten diet in non-coeliac adults may be driven by qualitative changes in dietary fibres.

## Abstract (web summary)

Gluten-free diets are increasingly common in the general population. Here, the authors report the results of a randomised cross-over trial involving middle-aged, healthy Danish adults, showing evidence that a low-gluten diet leads to gut microbiome changes, possibly due to variations in dietary fibres.

## Introduction

**Fig. 1:**
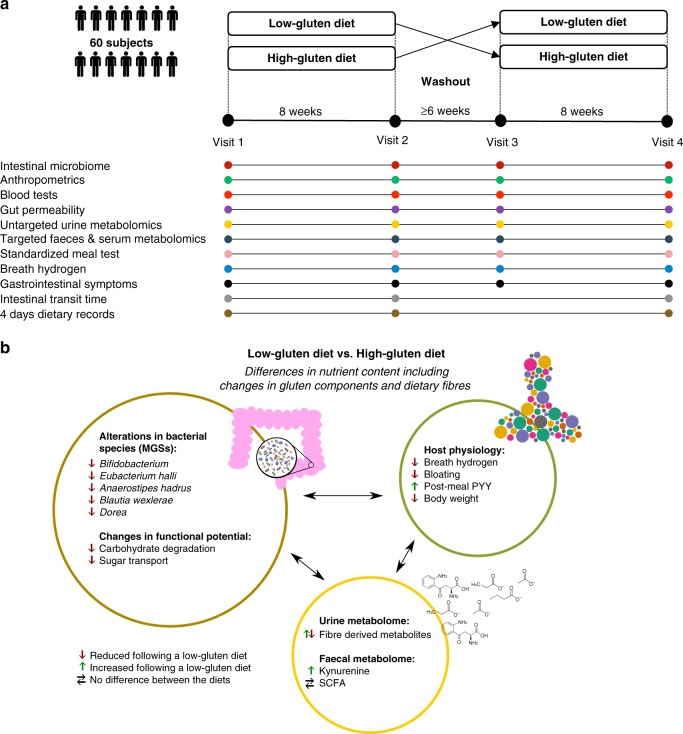
Experimental design, data overview and summary of the cross-over trial. **a** The study was a randomised, controlled, cross-over trial with two 8-week dietary intervention periods separated by a washout period of at least six weeks, comparing the effects of a low-gluten diet and a high-gluten diet on the gut microbiome (predefined primary outcome), untargeted urine metabolome and measures of host physiology^12^. Time points for data collections are indicated by circles in the lower part panel (**a**). **b** Effects of a low-gluten diet compared with a high-gluten diet on the intestinal microbiome, urine/faecal metabolome and markers of host physiology in apparently healthy adults. Measured variables that were found to be reduced (red arrow), increased (green arrow) or unchanged (black horizontal arrows) following the low-gluten diet intervention compared with the high-gluten diet intervention are listed. MGS metagenomics species, PYY peptide YY, SCFA short-chain fatty acids. The person icon and molecular structure images for the acetate anion, butyrate ion, propionate ion and kynurenine were obtained from Wikimedia Commons, released under public domain

Mechanistic and objective evidence on the effects of excluding gluten-rich food items for healthy adults is currently lacking, making the low-gluten diet highly debatable in public. Although not the sole component changed in a low-gluten diet, most discussion has centred on the dietary component gluten. Gluten is a major dietary component in wheat, rye and barley, and consists of proteins that are partially resistant to proteolytic digestion due to a high content of proline and glutamine^1,2^. Large gluten peptides including gliadin escape gastric digestion and accumulate in the small intestine, where they may interact with the immune system^3,4^, affect the intestinal permeability^5–7^, and modify the gut microbial activity^8,9^. However, beyond the reduction in gluten, a low-gluten dietary regime also entails a replacement of dietary fibres of gluten-rich cereals such as wheat, rye and barley with dietary fibres from other sources. Two short-term studies enroling 10 and 21 subjects based upon 16S rRNA gene profiling, respectively, have suggested that a gluten-free diet (GFD) changes the gut microbiome and immune function in healthy adults, however, with discrepant results^10,11^. Thus, it remains unsettled if a low-gluten diet affects the taxonomic and functional microbiome and host physiology of healthy individuals. Here we report the results of a randomised, controlled, cross-over trial encompassing 60 Danish adults without coeliac disease. We find that a low-gluten diet, in comparison with a high-gluten diet, induces changes in the composition and function of the gut microbiome (predefined primary outcome^12^), the urine metabolome and markers of host physiology (Fig. 1a, b).

## Results

### Cross-over intervention

To examine the impact of a low-gluten diet on the composition and function of the intestinal microbiome, urine metabolome and measures of host physiology, we undertook a randomised, controlled, cross-over trial with two 8-week dietary interventions comprising a low-gluten diet and a high-gluten diet, separated by a washout period of at least 6 weeks^12^. The trial was conducted from July 2012 to November 2013^12^. A total of 81 individuals were assessed for eligibility of which 18 did not meet the inclusion criteria^12^ and three declined to participate. Of notice, one excluded individual displayed elevated serum transglutaminase concentration (a marker of coeliac disease) and was excluded from the trial and referred for further clinical investigation. Sixty Caucasian Danish adults without coeliac disease, diabetes or any other self-known disorders were included^12^. They were between 22 and 65 years old, healthy by physical and biochemical examination, weight stable and had a body mass index (BMI) of 25–35 kg m^−2^ and/or increased waist circumference (≥ 94 cm for men and ≥ 80 cm for women). No study participants had a diagnosis of chronic disorders including a gastrointestinal disease. Study participants were randomly assigned to two groups: (1) undertaking either a low-gluten diet followed by high-gluten diet, or (2) high-gluten diet followed by low-gluten diet (Fig. 1a). In total, 51 participants completed the study and 54 participants had more than two visits and were included in the analyses (see baseline characteristics in Supplementary Table 1 and CONSORT flow diagram in Supplementary Fig. 1). During the two dietary interventions, study participants were asked to replace all cereal products with freely provided low-gluten or comparable gluten-rich dietary fibre-matched products of high-nutritional quality (Supplementary Table 2), which they were asked to consume ad libitum.

Overall, participants were highly compliant to both interventions, as documented in both food diaries (Supplementary Table 3) and according to measured fasting plasma alkylresorcinol concentrations, which were substantially reduced on the low-gluten diet compared with the high-gluten diet (Supplementary Table 4; *P* < 0.001, linear mixed model), providing objective evidence of individual compliance^13^. During the interventions, study participants consumed on average ± standard deviation 2 ± 2 g gluten per day (mainly from oats) during the low-gluten dieting period and 18 ± 6 g gluten per day (mainly from wheat and rye) during the high-gluten dieting period, in comparison to their habitual intake of 12 ± 4 g gluten per day (Supplementary Table 5). The habitual intake of gluten is comparable with a mean intake of 10.4 ± 4.4 g gluten per day in Denmark^14^, and the intake of gluten in the low- and high-gluten diets are in line with a previous study testing the effects of a low-gluten (2 g gluten per day) and high-gluten (16 g gluten per day) diet in patients with non-celiac gluten sensitivity^15^. Importantly, there was no difference between the two diet regimens in intake of total dietary fibre content. Intake of wholegrain cereals (wheat, rye and barley) was as expected lower in the low-gluten diet compared with the high-gluten diet (Supplementary Table 5; *P* < 0.001, paired t-test). There were no differences between the interventions in total energy or macronutrients intake, except for a slightly reduced protein intake during the low-gluten period (on average reduced with 7 g per day during the low-gluten period; Supplementary Table 5; *P* = 0.01, paired *t*-test). We compared the effects of the diets on changes in composition and functional potential of the gut microbiome, the urine metabolome, targeted serum and faeces metabolites and markers of host physiology using measurements of each variable taken at baselines (visit 1 and visit 3) and at end-points (visit 2 and visit 4) (Fig. 1a, b).

### A low-gluten diet alters the intestinal microbiome

**Fig. 2:**
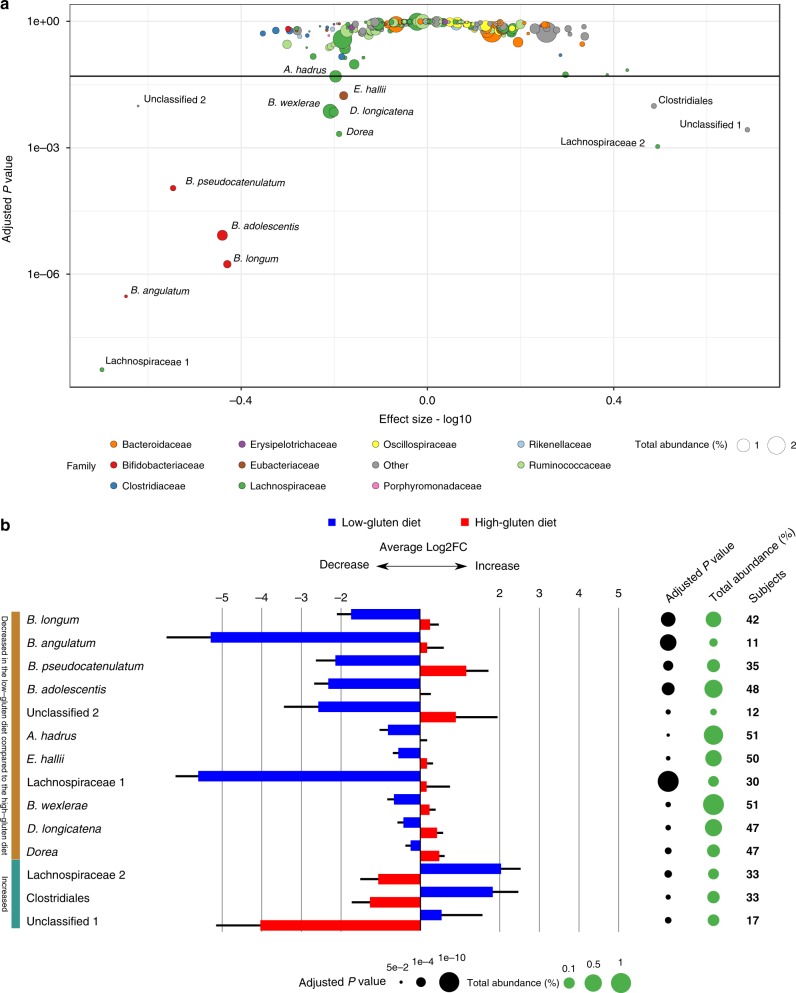
A low-gluten diet alters the composition of the gut microbiome. **a** Scatterplot of the statistical significance of the metagenomic species (MGSs) as assessed by a linear mixed model testing for the difference between the low-gluten and the high-gluten diets adjusted for age, gender, intestinal transit time, participant (*n* = 51) and carry-over effect. Adjusted *P* values are displayed on the *y*-axis (log10 scale) and the effect size (absolute values were log10 transformed) is on the *x*-axis. Points are sized according to the total abundance (%) and coloured according to the ten most abundant taxonomic families. The ‘Other’ category consists of the remaining families. The horizontal line represents an adjusted *P* value of 0.05 and the 14 species that changed significantly (FDR < 0.05) between the interventions are labelled with their full taxonomic annotation. Only species that could be annotated to family level and with abundance above 0.02% were included in the plot (255 species). **b** Bar chart of the 14 significant species showing the log2 fold change (means ± SEM) between baseline and after the low-gluten diet (blue bars) and high-gluten diet (red bars), respectively. The black circles are sized according to the negative log10 of the adjusted *P* values of comparison between the low-gluten and the high-gluten diet using a linear mixed model adjusted for age, gender, intestinal transit time, participant (*n* = 51) and carry-over effect. Green circles are scaled according the species abundance. The last column lists the number of participants in whom the given species were measured. Details on the individual species can be found in Supplementary Data 2

To estimate a potential impact of low-gluten versus high-gluten dieting on the gut microbiome, we studied a total of 208 individual whole-genome shotgun sequences of microbial DNA obtained from stool samples. On average, we obtained 6.7 Giga base-pairs (bp) per sample when including samples ranging from 3.7 to 13.6 Gbp (Supplementary Data 1). The microbial sequences were mapped to the integrated catalogue of reference genes of the human gut microbiome^16^ and genes were binned into metagenomic species (MGS; informal distinct microbial entities, from hereon called species) according to co-abundance variation across samples^17^. In total, 575 species were identified in at least ten individuals in this cohort. Of these species, the relative abundance of 14 bacterial species was altered during the low-gluten diet intervention compared with the high-gluten diet intervention (Fig. 2 and Supplementary Data 2; false-discovery rate (FDR) < 0.05, linear mixed model). Consistently, the abundance of four species of *Bifidobacterium* was diminished during the low-gluten diet (Supplementary Fig. 2). The substantial reduction in *Bifidobacterium* spp., both in terms of absolute and relative abundance, were confirmed by quantitative PCR (Supplementary Table 6). In addition, the low-gluten diet resulted in a decrease of a species annotated as *Dorea longicatena* and another species of *Dorea*, one species of *Blautia wexlerae*, two species of the *Lachnospiraceae* family, and two butyrate-producing bacteria *Anaeostipes hadrus* and *Eubacterium hallii*, in comparison with the high-gluten diet. At the same time, an unclassified species of unknown taxonomic origin, an unclassified species of *Clostridiales* and an unclassified species of *Lachnospiraceae* increased during the low-gluten diet intervention compared with the high-gluten diet intervention. Notably, we did not find any changes in alpha- and beta-diversity (Supplementary Fig. 3).

**Fig. 3:**
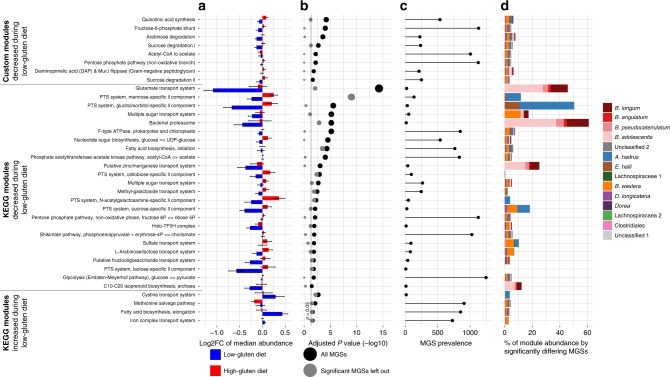
A low-gluten diet alters the functional potential of the gut microbiome. **a** Microbial genes annotated to Kyoto Encyclopedia of Genes and Genomes (KEGG) orthologs (KOs) were grouped into KEGG modules and manually curated (customised) modules. The bar chart display the median log2 fold change (median ± SEM) of all individual KOs within a module when comparing the relative abundance at baseline to the abundance following the low-gluten diet (blue bars) or the high-gluten diet (red bars), respectively. **b** Dot plot of the negative log10 of the adjusted *P* value from the linear mixed model comparing changes in the abundance of modules induced by the low-gluten diet with the changes induced in the high-gluten diet (black dots) adjusting for age, gender, intestinal transit time, participant (*n* = 51) and carry-over effect. The same analysis was carried out while removing the significant MGSs from the data (grey dots) to elucidate their contribution to the significance. All effect sizes and SEM for each KO can be found in Supplementary Data 3 and 4. **c** Prevalence of the module across the 1264 MGSs identified from the IGC catalogue^16,17^. A module was assessed to be present or partially present in a MGS when at least two KOs from the module were detected in the MGS**. d** Bar plot showing the fraction of the total abundance of a module contributed by each significantly different MGS in per cent. (Supplementary Data 2)

To explore changes in the functional capacity of the intestinal microbiome following low-gluten as compared with the high-gluten dieting, all microbial genes annotated to prokaryotic Kyoto Encyclopedia of Genes and Genomes (KEGG) orthologies (KOs)^18^ were tested individually, when grouped into KEGG modules^19^ and when grouped into customised reference modules^20^, respectively. We identified 88 KOs and 37 modules that changed following the low-gluten diet period compared with the high-gluten diet intervention (Fig. 3 and Supplementary Data 3 and 4; FDR < 0.05, linear mixed model). In particular, the abundance of modules associated with carbohydrate metabolism (i.e. arabinose degradation, pentose phosphate pathway, phosphate acetyltransferase-acetate kinase pathway and fructose-6-phosphate shunt) and uptake of carbohydrates (L-arabinose/lactose transport system, phosphotransferase systems (PTS) and other sugar transport systems) was diminished following the low-gluten dieting compared with the high-gluten dieting. This suggests a change in bacterial carbohydrate degradation as a response to the dietary intervention. Furthermore, abundance of modules associated with bacterial transport of glutamate, zinc/manganese and sulphate was diminished, whereas abundance of modules associated with transport of cysteine and iron was increased following the low-gluten diet compared with the high-gluten diet period. A majority of the modules showed subtle changes in the functional potential (Fig. 3a, b), high prevalence in the species (Fig. 3c) and the significantly different species comprised a minor percentage of the total functional potential (Fig. 3d). However, leaving out the significantly different species showed that they contributed considerably to the observed significant changes in functional potential for multiple of the modules (Fig. 3b). In summary, these findings demonstrate that low-gluten dieting changes the gut microbiome composition and functional potential in healthy adults.

### A low-gluten diet changes the intestinal fermentation

**Fig. 4:**
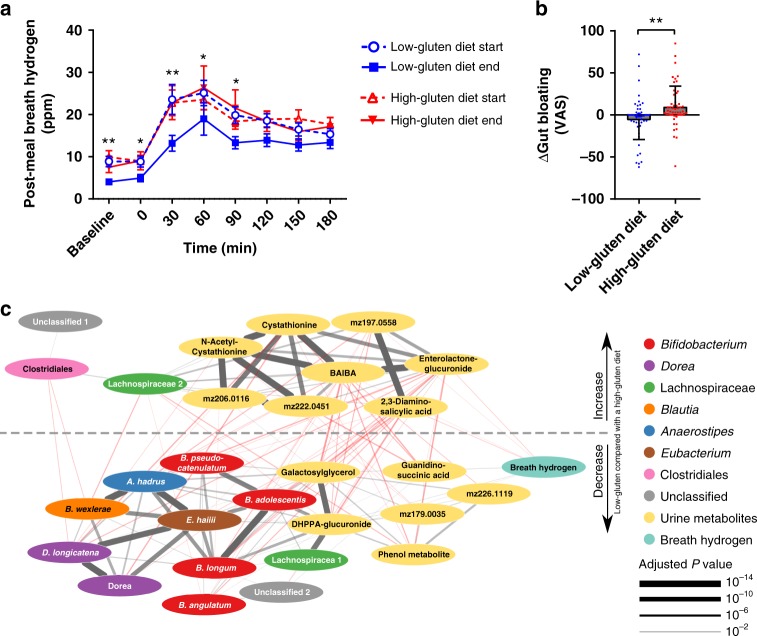
A low-gluten diet affects measures of intestinal fermentation. **a** Breath hydrogen levels following the same standardised meal at all four visits (low-gluten diet start, open blue circles; low-gluten diet end, blue squares; high-gluten diet start, open red triangle; high-gluten diet end, filled red triangle). Data are shown as means ± SEM (n = 51-57). **b** Plot showing changes in gut bloating as assessed by visual analogue scale (VAS) following the low-gluten diet (blue circles) compared with the high-gluten diet (red squares). Data are shown as means ± SEM (*n* = 52–53). Changes were assessed by a linear mixed model adjusting for age, gender and intestinal transit time. *P < 0.05, **P < 0.01. **c** Linear regression network of breath hydrogen levels and the abundance of bacterial species and concentrations of urine metabolites which are significantly responding to the dietary interventions using a linear mixed model adjusted for gender, age and participant (*n* = 49) (Supplementary Data 5). The dotted line separates the features that were decreased and increased, respectively, when comparing the low-gluten and high-gluten periods. Significant (FDR < 0.05) positive associations are indicated with grey lines; negative associations with red lines. Thickness of lines indicates the significance level. Nodes are coloured according to type; breath hydrogen (cyan), urine metabolites (yellow), *Bifidobacterium* (red), *Dorea longicatena* (purple), *Blautia wexlerae* (orange), *Eubacterium hallii* (brown), *Lachnospiracaea* (green), *Anaerostipes* (blue), Clostridiales (pink) and Unclassified (grey). *m/z* refers to the mass-to-charge ratio of a given unidentified urine metabolite. BAIBA β-aminoisobutyric acid, DHPPA 3,5-dihydroxy-hydrocinnamic acid

We found a reduction in both fasting and postprandial hydrogen exhalation after an identical standardised test meal following low-gluten dieting compared with high-gluten diet dieting (Fig. 4a and Supplementary Table 7; *P* *<* 0.0001, linear mixed model). In addition, participants reported improved postprandial well-being after the standardised meal following the low-gluten diet compared to the high-gluten diet (Supplementary Fig. 4). Whereas the change in breath hydrogen was primarily driven by the low-gluten diet, the change in postprandial well-being was unexpectedly primarily driven by the high-gluten diet. However, the reduction in breath hydrogen was convergent with participants reporting less bloating following the 8-week low-gluten intervention compared with the 8-week high-gluten intervention (Fig. 4b). Together these observations suggest an altered intestinal fermentation in accordance with the changes in bacterial modules associated with carbohydrate metabolism (Fig. 3). Indeed, several differences in carbohydrate composition were found between diets including higher levels of galactose, rhamnose, mannose, and galacturonic acid and lower levels of arabinose and xylose in the low-gluten diet compared with the high-gluten diet (Supplementary Fig. 5a). These nutritional changes were in agreement with a reduced bacterial arabinose/lactose transport potential following the low-gluten dieting (Fig. 3). There was no differences in the total amount of dietary fermentable, oligo-, di-, and monosaccharides and polyols (FODMAP) (Supplementary Fig. 5b) or in intake of resistant starch (Supplementary Table 2) between the two diets. Yet, qualitative differences were observed, such as lower levels of fructooligosaccharides and mannitol/sorbitol and higher levels of lactose in the low-gluten diet. In support of a changed intestinal fermentation, breath hydrogen concentrations were negatively associated with gut metabolic modules related to methanogenesis (Supplementary Table 8). The latter comprises reduction of CO_2_ to CH_4_ using H_2_ or formate as electron donors^21^ occurring alongside proteolytic degradation following prolonged intestinal transit times^22^. We did not find, however, any changes in intestinal transit time (Supplementary Table 7).

To further explore changes in intestinal fermentation, we performed untargeted metabolic profiling of urine sampled during the standardized meal tests by gas chromatography mass spectrometry (GC-MS) as well as by ultra-performance liquid chromatography mass spectrometry (UPLC-MS). We found lower concentrations of wheat-derived compounds (3,5-dihydroxyhydrocinnamic acid-glucuronide and galactosylglycerol) during the low-gluten intervention in comparison with the high-gluten intervention. In contrast the urinary concentration of a host-microbial co-metabolite of lignan degradation (enterolactone-glucuronide) was increased during the low-gluten intervention (Supplementary Table 9; FDR < 0.05, linear mixed model), suggesting an altered dietary fibre degradation upon reduction in gluten-rich food items during the low-gluten diet and related changes in the gut microbiome.

To identify correlations between breath hydrogen levels and changes in the gut microbiome and urine metabolome, we developed a co-occurrence network of breath hydrogen and the bacterial species and urine metabolites that responded to the dietary interventions (Fig. 4c and Supplementary Data 5). Breath hydrogen was positively associated with the wheat-associated urine metabolites and *B. longum* and negatively associated with urine enterolactone-glucuronide, substantiating that differences in composition of dietary fibres between the two diet regimens resulted in a changed colonic fermentation. The network analysis identified the lactate-utilising, butyrate-producing *Eubacterium hallii* as a key driver species, which was associated with the lactate-producing *Bifidobacterium*, as well as the hydrogen-producing *Dorea longicatena* and the hydrogen-consuming, acetate-producing *Blautia*. Furthermore, the network analysis highlighted associations between the wheat-associated urine metabolites and the *Bifidobacterium* species, suggesting that the reduction in *Bifidobacterium* abundance following the low-gluten diet intervention was associated with the diminished intake of wheat. Likewise, the abundance of microbiome modules associated with uptake and degradation of mannose, sucrose and arabinose was positively associated with wheat-associated urine metabolites (Supplementary Fig. 6 and Supplementary Table 10). Collectively, these results suggest that a changed gut microbiome and altered fermentation resulting from qualitative differences in dietary fibre composition may explain the reduced breath hydrogen and reduced bloating following the low-gluten diet.

### A low-gluten diet results in weight loss

**Fig. 5:**
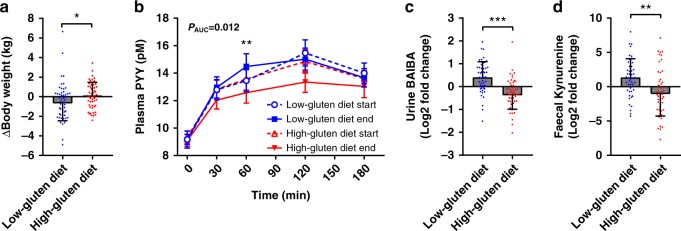
Low-gluten dieting affects markers of host metabolism. **a** Plot showing participants’ changes in body weight following the low-gluten (blue circles) and high-gluten (red squares) periods. **b** Plot showing participants’ plasma concentrations of peptide YY (PYY) following a standardised meal at all four visits (low-gluten diet start, open blue circles; low-gluten diet end, blue squares; high-gluten diet start, open red triangle; high-gluten diet end, filled red triangle). **c** Plot showing log2 fold changes in participants’ urine concentrations of β-aminoisobutyric acid (BAIBA) and **d** faecal concentrations of kynurenine following the low-gluten (blue circles) and high-gluten (red squares) diet, respectively. Data are shown as means ± SEM, *n* = 50–54. Changes were assessed by a linear mixed model adjusting for age, gender and intestinal transit time. *P < 0.05, **P < 0.01, ***P < 0.001. AUC area under the curve

We did not find any differences in measures of glucose and lipid metabolism (Supplementary Table 7). However, we found a decrease in body weight, on average 0.8 ± 0.3 kg, following the low-gluten dieting for 8-week compared with the high-gluten diet period (Fig. 5a; *P* *=* 0.012, linear mixed model). We also demonstrated an increase in postprandial plasma concentrations of peptide YY (PYY) in response to the standardised meal after the low-gluten intervention compared with the high-gluten intervention (Fig. 5b and Supplementary Table 7; *P*_*AUC*_ = 0.012, linear mixed model). PYY is a gut hormone released into the circulation in a nutrient-dependent manner and is known to reduce appetite^23^. However, we did not observe any differences in total energy intake during the two interventions (Supplementary Table 5). There were no changes in levels of the proximal incretin hormone gastric inhibitory peptide (GIP), and no changes in the distal gut hormone glucagon-like peptide 2 (GLP-2), a regulator of gut mucosal adaptation and growth; GLP-2 is known to be secreted in comparable amounts in parallel to the appetite regulating hormone GLP-1, and it may therefore be assumed that also GLP-1 secretion was unchanged^24^ (Supplementary Table 7). Together, these findings suggest that a different mechanism is responsible for the weight loss. Colonic short-chain fatty acids (SCFA), synthesized by the gut microbiota during fibre fermentation, are known to increase plasma PYY levels, fat oxidation and energy expenditure in overweight men^25^. However, we did not observe any associations between changes in fasting or postprandial plasma PYY concentrations and changes in bacterial modules associated with SCFA biosynthesis potentials (Supplementary Table 8) or in faecal and serum concentrations of SCFA (Supplementary Table 11). Fasting plasma PYY concentrations have been negatively associated with various markers of adiposity and resting metabolic rate in humans^26^, and long-term elevated PYY concentrations are associated with enhanced thermogenesis in mice^27^. Among the differing urine metabolites, β-aminoisobutyric acid (BAIBA) was increased following the low-gluten diet compared with the high-gluten diet period (Fig. 5c and Supplementary Table 9; FDR < 0.05, linear mixed model). BAIBA induces browning of white adipose tissue and increases hepatic fat oxidation^28^. Changes in urine BAIBA concentrations were, however, not associated with the bacterial pyrimidine degradation module (Supplementary Table 8), which contains bacterial genes involved in degradation of thymine into BAIBA, suggesting that the observed changes in urine BAIBA were not related to changes in the intestinal microbiome. Rather they might be directly related to other effects of the low-gluten diet on host metabolism. Together, the increased urine concentrations of BAIBA and the elevated postprandial plasma levels of PYY suggest that intake of the low-gluten diet modulated energy homoeostasis by changing thermogenesis or fat oxidation. To explore these hypotheses, we performed targeted metabolomics quantifying fatty acids, acyl-carnitines (transport fatty acids into the mitochondria for breakdown), and BAIBA in serum. Besides a significant increase in serum linoleyl-carnitine following the low-gluten diet compared with the high-gluten diet, these metabolites were not changed (Supplementary Table 12), suggesting unaltered fat oxidation. Further exploring possible reasons for the observed weight loss, we targeted metabolites associated with the microbiota-gut-brain axis including serotonin, kynurenine, glutamate, γ-aminobutyric acid^29,30^ in faeces and serum. Analyses of these metabolites did reveal a significant increase in faecal kynurenine concentrations following the low-gluten diet intervention compared with the high-gluten diet intervention (Fig. 5d and Supplementary Table 12; *P* = 0.005, linear mixed model). Compared with healthy controls, coeliac disease patients adhering to a GFD have been reported to have lower serum concentrations of aromatic amino acids including tryptophan, the substrate for kynurenine^31^. Therefore, we quantified serum and faeces concentrations of the aromatic amino acids and their derivatives. Since concentrations of tryptophan and microbial tryptophan catabolites were unaltered (Supplementary Table 12), the observed increase in kynurenine faeces concentration following the low-gluten diet suggested altered microbiota tryptophan degradation pathways rather than being a mere consequence of substrate availability. Indeed, targeted metagenomic module analyses revealed a proportional decrease in the potential of the tryptophan to serotonin synthesis pathway (Supplementary Data 6 and Supplementary Fig. 7) following the low-gluten diet. Moreover, we found the ratios of the proportional abundances of their respective production pathways and faecal concentrations to correlate (Supplementary Fig. 8a; Spearman rho = 0.20; *P* = 0.004; Supplementary Table 13), suggesting a balance between both tryptophan conversion routes (Supplementary Fig. 7**)**. In rodents, kynurenic acid, a downstream product of kynurenine, has been reported to enhance adipose tissue thermogenesis through activation of G protein-coupled receptor Gpr35^32^, which is also highly expressed in the gastrointestinal tract^33^. Here, we observed faecal kynurenine concentrations to be positively associated with urine BAIBA levels (Supplementary Fig. 8b; Spearman rho = 0.26; *P* = 9.2E–05**)**, indicating a potential role of the colon microbial production in fat browning.

### A low-gluten diet has subtle effects on the immune system

To determine the potential impact of a low-gluten diet on immune and inflammatory host responses, we assessed systemic inflammatory markers as well as ex vivo lipopolysaccharide (LPS)-induced cytokine responses in whole-blood of study participants. We did not find any changes in concentrations of systemic inflammatory markers in serum (i.e., C-reactive protein (CRP), interleukin (IL)-6 or tumour necrosis factor alpha (TNF-α); Supplementary Table 7) or in counts of immune cell populations in blood (i.e. leucocytes, lymphocytes, neutrophils, monocytes; Supplementary Table 7). Neither did we find any changes in markers of intestinal inflammation (i.e. fasting plasma citrulline and faecal calprotectin; Supplementary Table 7) nor in intestinal permeability as measured by fasting serum zonulin and urinary excretion of lactulose and mannitol (Supplementary Table 7). Of notice, ex vivo LPS-induced stimulation of whole-blood showed, however, reduced release of the pro-inflammatory, inflammasome-related cytokine IL-1β following the low-gluten diet intervention compared with the high-gluten diet period (Supplementary Table 7; *P* = 0.035, linear mixed model). None of the other serum concentrations of non-inflammasome-related, pro-inflammatory mediators such as IL-6, TNF-α and interferon gamma (IFN-γ) were changed. These findings suggest a selectively reduced activation of the inflammasome response following the low-gluten diet intervention compared with the high-gluten diet period. Intriguingly, we demonstrated a positive association between the abundance of the bacterial Lipid A synthesis module (present in all Gram-negative bacteria) and LPS-induced release of IL-1β from whole-blood (Supplementary Table 8). Collectively, these results suggest that a low-gluten diet confers a selectively reduced activation of the inflammasome response.

## Discussion

An overview of the outcome of this randomised, controlled, cross-over trial with two 8-week dietary intervention periods comparing the effects of a low-gluten diet and a high-gluten diet is given in Fig. 1.

We showed that a low-gluten diet in apparently healthy adults changed the primary trial endpoint, the gut microbiome composition and functional potential. Among the 14 bacterial species which changed between the two dietary regimens, particularly the relative abundance of *Bifidobacterium* species was consistently diminished following adherence to the low-gluten dietary regimen. This finding is in agreement with a microbiota gene marker study involving 10 healthy adults showing that a shift to a GFD for four weeks resulted in decreased proportions of *Bifidobacterium*^10^, as well as with reports of lower abundance of bifidobacteria in celiac disease patients following a GFD^34,35^. In addition, practicing a low FODMAP diet diminishes the abundance of bifidobacteria in patients with IBS concurrent with relief of gastrointestinal symptoms^36–38^. These interventions generally reduce intake of wheat or exclude wheat, suggesting a close relationship between wheat intake and the abundance of bifidobacteria in adults. This aligns with recent studies showing that healthy populations living traditional lifestyles have low or absent faecal abundance of bifidobacteria compared with the intestinal ecosystems of individuals in industrialised parts of the world^39,40^. Thus, the abundance of bifidobacteria in adults living a Western lifestyle may to a large extent reflect intake of diets enriched in wheat.

In parallel, we observed a reduction in butyrate-producing *E. hallii* and *A. hadrus* as well as in the hydrogen-producing *Dorea* and the hydrogen-consuming, acetate-producing *Blautia*, following the low-gluten diet compared with the high-gluten diet. These interrelated species were positively associated, which is consistent with reports on cross-feeding between *Bifidobacterium* and butyrate-producing bacteria^41–43^, with *Blautia’* ability to produce acetate and utilise hydrogen during fibre fermentation^41,44,45^ and with the ability of *Dorea longicatena* to produce hydrogen^46^. Several in vivo and in vitro studies have shown bifidogenic effects and stimulation of butyrate-producing colon bacteria^47–51^ by arabinoxylan and arabinoxylan-oligosaccharides, abundant non-starch polysaccharides of cereal grains^52^. Indeed, the fibre composition analysis of the two intervention diets showed lower concentration of arabinose in the low-gluten products compared to the high-gluten products. This was in agreement with changes in the functional potential of the microbiome upon the low-gluten dieting. A module representing a L-arabinose/lactose transport system and a custom module representing arabinose degradation, which converts L-arabinose to L-ribulose-5-phosphate, were significantly reduced during a low-gluten diet. Further, L-ribulose-5-phosphate is utilized by the non-oxidative phase of the pentose phosphate pathway, which was also significantly reduced during the low-gluten period compared to the high-gluten period. This suggests that the replacement of grain-derived fibres of wheat, barley and rye with dietary fibres of other sources during the low-gluten diet intervention caused the observed changes in the intestinal microbiome. Importantly, in accordance with the diets being matched for dietary fibres, we did not during our 8-week intervention observe any changes in faecal and serum SCFA. Furthermore, we did not find any health implications associated with the reduction in *Bifidobacterium* and butyrate-producing species following the low-gluten diet, although the long-term health consequences remain unknown.

Despite the unchanged concentrations of SCFA concentration in serum and faeces, we observed a reduction in both fasting and postprandial hydrogen exhalation following the low-gluten diet intervention and multiple changes in urine metabolites reflecting a changed intestinal fermentation. In line with this, a previous study reported that fasting breath hydrogen concentrations were significantly lower in coeliac disease patients on a GFD compared with untreated coeliac disease patients^53^. Likewise, a low FODMAP diet has been reported to reduce breath hydrogen and ameliorate gastrointestinal symptoms compared with a high FODMAP diet^15,37^. To which extent the concurrent improvements in well-being and bloating following the low-gluten diet intervention as compared with the high-gluten diet period, were prompted by changes in the intestinal microbiome and fermentation, or were due to psychological (placebo) effects remain unresolved.

No effects on glucose and lipid metabolism were found. However, despite unaltered self-reported energy intake by study participants, the low-gluten diet was temporarily linked with a significant weight loss. This is in line with two studies in mice fed a gliadin-enriched, high-fat diet showing an increase in body weight and adiposity^54,55^. Still, other studies in mice show no effect of gluten on body weight^3,56,57^. Based on the observed increase of plasma PYY and urinary BAIBA concentrations following the low-gluten diet, we hypothesized that the reduced body weight induced by low-gluten intervention might in part be mediated by an increased thermogenesis. Recent studies in mice have indicated that increased intake of gluten may increase hepatic lipid accumulation^3^, reduce the thermogenic capacity of adipose tissue^55^ and the size of adipocytes^3^. Here we found that faecal concentrations of kynurenine were increased following the low-gluten diet and associated with urine BAIBA, raising the intriguing possibility that kynurenine, via the downstream product of kynurenic acid, enhance thermogenesis^32^ through activation of Gpr35 in the gastrointestinal tract^33^. Obviously, further interventions in humans are warranted to specifically delineate whether intake of a low-gluten diet modulates energy homoeostasis.

Our analyses showed that the low-gluten diet had no effect on circulating white blood cell counts or markers of systemic inflammation in unstimulated blood or on measures of intestinal inflammation. Likewise, no effects were seen on intestinal permeability markers. We did, however, notice that LPS-induced stimulation of whole-blood showed that immune cells had reduced capacity to produce the pro-inflammatory, inflammasome-related cytokine IL-1β^58^ following the low-gluten diet period. Similarly, a previous study reported that production of pro-inflammatory cytokines by peripheral blood mononuclear cells stimulated with faecal water was reduced after a GFD^10^. As the inflammasome-directed response also takes place in intestinal cells in a similar manner, and is regarded as an important regulator of intestinal homoeostasis^59,60^, these findings might point to a yet undescribed impact of a low-gluten diet on the immune system that in future studies will need further clarification.

In conclusion, an 8-week low-gluten diet intervention in healthy middle-aged adults induced changes in the intestinal microbiome and fermentation of complex carbohydrates as mirrored in changes of the urine metabolome and reduction in breath hydrogen. Although the generalizability to other populations is to be determined as gluten consumption differs in Western populations^61,62^, the changes in colonic microbial composition and fermentation suggest that the effects of a low-gluten diet in healthy middle-aged adults may to some extent be driven by qualitative changes in dietary fibres upon reduction of gluten-rich food items rather than by the reduction of gluten intake itself.

## Methods

### Trial design

This was a randomised controlled (1:1) cross-over trial composed of two 8-week dietary interventions comprising a low-gluten diet or a high-gluten diet, separated by washout period for at least six weeks (range 6–23 weeks, median of 8 weeks) with habitual diet. The trial design, intervention modes and primary and secondary outcomes have been reported in a previous paper^12^ and registered at www.clinicaltrials.gov (NCT01719913). The trial was conducted from July 2012 to November 2013^12^.

### Participants

Participants were recruited from the general population studies “Health 2008” and “Health 2010”, established at the Research Center for Prevention and Health (RCPH) at Glostrup University Hospital in Copenhagen, Denmark^63^ and through the webpage www.forsogsperson.dk and advertisements in local newspapers. Participants were non-diabetic, lean, overweight or obese adults who were healthy by self-report and aged 22–65 years. Importantly, they did not suffer from coeliac disease or other gastrointestinal diseases. In order to detect latent coeliac diseases, levels of serum Immunoglobulin(Ig)A and IgG transglutaminase were measured at the first examination day. In case values exceeded the acceptable maximum (> 8 units per mL for IgA and >10 units per mL for IgG) participants were excluded from the study and referred to own general practitioner. Further eligibility criteria have been published elsewhere^12^. Exclusion criteria included antibiotic treatments (< 3 months prior to study start), intake of pre- or probiotic supplements (<1 month prior to study start), medically prescribed diet and intense physical activity (>10 h per week)^12^. Data on participants’ physiological traits and smoking habits are available in Supplementary Table 1.

The study was led by the Novo Nordisk Foundation Center for Basic Metabolic Research, Faculty of Health and Medical Science, University of Copenhagen and conducted at the Department of Nutrition, Exercise and Sports at the University of Copenhagen, Denmark. The Ethical Committee of the Capital Region of Denmark approved the trial (H-2-2012-065), which was run in accordance with the Helsinki declaration and endorsed by the Data Protection Agency (2007-54-0269). All individuals gave written informed consent before participating in the study.

### Interventions

The aim of the dietary interventions was to limit the daily gluten consumption considerably in the low-gluten period (~2 g per day) and to increase it in the high-gluten period (~20 g per day). For comparison, in the national survey of dietary habits, Danish adults (*n* = 1494, 20–75 years) had a mean total gluten intake of 12.0 ± 4.6 g per day in men and 9.0 ± 3.4 g per day in women^14^. During the two dietary interventions participants were provided with a selection of low-gluten or high-gluten products of high nutritional values and instructed to replace *all* cereal products from their habitual diet with the study dietary products and to consume these products ad libitum (Supplementary Table 2). Each participant was randomly assigned to start on either the low-gluten diet or the high-gluten diet. Participants were encouraged to contact the study staff if they experienced any adverse health-related implications of the dietary interventions. The outline of the trial is shown in Fig. 1.

### Overview of protocol measures

The primary endpoint was an altered gut microbiota composition and functional potential during consumption of a low-gluten compared with a high-gluten diet as measured by shotgun sequencing-based metagenomics analyses of microbial DNA isolated from faecal samples and sequenced applying deep metagenomics sequencing^12^. Secondary outcomes^12^ included body weight, waist circumference, sagittal diameter, fasting concentrations of plasma glucose, serum insulin, serum C-peptide plasma GIP, serum triglycerides (TAG), serum total cholesterol, serum high-density lipoprotein (HDL) cholesterol, serum low-density lipoprotein (LDL) cholesterol, serum alanine-aminotransferase (ALAT), serum aspartate aminotransferase (ASAT), serum CRP, serum IL-6, serum TNF-α, whole-blood haemoglobin, white blood cells, whole-blood lymphocytes, mix of whole-blood monocytes, eosinophils as well as basophils, whole-blood neutrophils, serum IL-6, serum TNF-α, serum zonulin, plasma citrullin, homoeostatic model assessment for insulin resistance (HOMA-IR), whole-blood glycated haemoglobin (HbA1c) and targeted serum and faeces metabolites. In addition, during a standardized meal test measurement of postprandial responses of plasma glucose, serum insulin, plasma GLP-2, plasma peptide YY (PYY), plasma free fatty acids (FFA), exhalation of H_2_, untargeted UPLC-MS and GC-MS urine metabolomics, urine lactulose and mannitol excretion. Further examinations included measurement of faecal calprotectin, intestinal transit time, average number of defaecations over the last week, Bristol stool scale estimates of stool consistence, well-being and gastrointestinal comfort indicators (bloating), and ex vivo cytokine production in LPS-stimulated whole-blood.

### Sample size

Estimations were based on 85% statistical power to detect a difference of 0.4 standard deviation in metabolic quantitative traits, based on previous observations from the MetaHit study^64^. It was estimated that 51 individuals were needed, but to allow for a 15% dropout after randomization, a total of 60 participants were invited for participation. Additionally, based on observed standard deviations for the MGSs changing during the low-gluten and high-gluten interventions, we concluded that the number of included subjects was adequate to provide evidence of a changed intestinal microbiome after a low-gluten diet compared with a high-gluten diet.

### Randomisation

The random allocation sequence was generated by an investigator without contact to the participants (www.randomization.com). Details of the type of randomisations and restrictions such as blocking and block size have been published previously^12^. The random allocation sequence was implemented by the dietician using a list of participant IDs matched with allocated sequences.

### Blinding

The participants and the investigators involved in outcome assessment were blinded until the first examination day. Thereafter, blinding was not possible due to the nature of the intervention. However, blinding of the allocation sequence was maintained during sampling of biological materials and initial steps of bioinformatics and statistical analyses.

### Anthropometrics

On the four examination days, before and after each intervention, participants met in the morning after an overnight fast of ≥10 h and absenting from physical activity and alcohol consumption for ≥ 24 h. In addition, participants were instructed to avoid smoking and tooth brushing in the morning of the examination days. Prior to determination of body weight, participants were asked to empty their bladder and to wear only underwear or light clothing. Body weight was determined and registered to the nearest 0.05 kg (Lindell Tronic 8000, Digital Medical Scale, Copenhagen, Denmark). At the first examination day only, height was measured with a wall-mounted stadiometer while the participants were barefooted and it was registered to the nearest 0.1 cm (Hultafors, Sweden). Waist circumference was measured twice using a flexible measuring tape (Meterex, Lagenfeld, Germany) at the point of the umbilicus after an exhalation and was registered to the nearest 0.5 cm. Sagittal abdominal diameter was measured twice using an abdominal calibre (Holtain-Kahn Abdominal Caliper, Crosswell, UK) at the umbilicus level after an exhalation with participants lying on a flat bed with the legs bent and was registered to the nearest 0.1 cm.

### Biochemical analyses of fasting blood samples

Blood samples were drawn via an intravenous cannula in the participants’ antecubital vein at all four examination days. Shortly after collection, the blood samples were stored in ice water, separated into serum and plasma, and immediately stored at −80 °C until analyses. All blood sample analyses were performed in one batch at the end of the study to ensure low variability.

Plasma glucose, whole-blood HbA1c and serum TAG, total-, LDL-, and HDL-cholesterol, ALAT and ASAT were analysed using automated, enzymatic, colorimetric assay on ABX Pentra 400 chemistry analyser (ABX Pentra, Horiba ABX, Montpellier, France). The coefficients of variation (CV) for these analyses were between 1.3 and 7.2%.

Serum insulin and C-peptide were measured by a chemiluminescent immunometric assay (Immulite 1000; Siemens Medical Solutions Diagnostics, Los Angeles, USA). CV was < 5% for both. HOMA-IR was calculated according to Wallace et al.^65^ as insulin resistance = glucose in mmol L^−1^ × insulin in pmol L^−1^ × 135^−1^. Serum CRP was measured after a 1000× dilution in a high-sensitivity single-plex assay (MesoScale Discovery®, Gaithersburg, MD, USA) using the Sector Imager 2400A (MesoScale Discovery®). The lower limit of detection was 4.3 pg mL^−1^. Blood counts of total haemoglobin, leucocytes, neutrophils, lymphocytes, and others immune cells (including monocytes, mast cells, basophils and eosinophils) were obtained using a Sysmex KX-21 automated haematology analyser (Sysmex America Inc., Lincolnshire, Illinois, USA). Serum IL-6 and TNF-α were measures by high-sensitivity enzyme-linked immunosorbent assays (ELISA) (R&D systems, Minneapolis, Minnesota, USA, HSLB00C, HS600B, and HSTA00D, with detection limits: 0.15 pg mL^−1^ and 0.5 pg mL^−1^, respectively). The CV% was 3.6% and 5.2%, respectively.

Plasma citrulline, a marker of enterocyte capacity and mass, was measured using ultra-performance liquid chromatography tandem mass-spectrometry of acetonitrile-derived supernatants originally validated and described elsewhere with a CV% of 2.0–4.3^66^. Serum zonulin, a marker of tight junction regulation^67^, was measured using IDK Zonulin ELISA kit (Immundiagnostik AG, Bensheim, Germany). The CV% was 7.5%.

Plasma alkylresorcinols, markers of wholegrain wheat, rye and quinoa intake, were analysed using normal-phase ultra-performance liquid chromatography tandem mass-spectrometry^68^.

### Dietary intake assessment

Participants completed a 4-day pre-coded dietary record, developed and used at the National Food Institute at the Technical University of Denmark^69,70^ to assess the habitual dietary intake in the national dietary survey. The record was filled out on two weekdays and two weekend days at study start and at the end of both interventions. Daily intake of total energy, macronutrients, certain food components and food groups were calculated (habitual diet only without estimates from intake of study products). The gluten content of the study dietary products was calculated based on data from the food database at the Danish Food Composition Databank containing 1049 food items^71^.

### Dietary compliance

Participants recorded a study diary, in which they registered daily consumption (amount and type) of study dietary products throughout both interventions as well as any deviations from the dietary instructions in the diary. A trained dietician conducted a follow-up telephone call every second week prior to home delivery of study dietary products, focusing on consumption of study dietary products and adherence to the dietary regimens in general. The diary was used as an objective measure of compliance to the intervention and to calculate absolute consumption of study dietary products. In addition, the concentration of alkylresorcinols in the blood was analysed as a measure of compliance, since these are biomarkers of grains^13^. The study diary was also used for noting any illness and use of antibiotics, during the interventions.

### Dietary fibre composition of the two diets

The dietary fibre composition of the two diets were determined by measuring the monosaccharide composition of a representative meal of each distinct diet, the resistant starch composition of the dietary study products, and the FODMAP (fermentable oligosaccharides, disaccharides, monosaccharides and polyols) composition of the provided low-gluten and the high-gluten study products. The details are available in Supplementary Methods.

### Faecal sample collection and DNA extraction

Faecal samples were collected in the morning of the four examination days and immediately stored at 5 °C for a maximum of 24 h before equal volume of sterile water was added and the sample was homogenised. The homogenised sample was aliquoted to cryotubes, and stored at −80 °C. Microbial DNA was extracted from the faecal samples as previously reported^72^.

### Metagenomic sequencing and quantitative PCR

The community DNA from all faecal samples was sequenced by metagenomics sequencing. In addition, quantification of *Bifidobacterium* spp. and total bacterial load in all faecal samples was performed by quantitative PCR. Details are available in Supplementary Methods.

### Ex vivo cytokine production after stimulation with LPS

Within 30 min of blood sampling, 50 μL of whole-blood was LPS stimulated in triplicates after having been diluted 1:10 in RPMI medium (LONZA, BE12-167F) supplemented with LPS (Sigma-Aldrich, L2645-1MG) in a final concentration of 1 μg mL^−1^. Samples were incubated for ~24 h at 37 °C and 5% CO_2_ in order to determine ex vivo cytokine production. After incubation supernatants were harvested and stored at −80 °C until ELISA measurements of IL-1β, IL-6, TNF-α and IFN-γ (R&D Systems, DY201, DY206, and DY210, respectively)^73^. The CV% was 3.6–5.2%.

### Standardised meal test

On the four examination days, participants were lying and resting for at least 10 min before blood samples were drawn in the at least 8 h fasting state (*t* = 0 min) and postprandial (*t* = 30, 60, 120 and 180 min) after consumption of the same standardised breakfast, no matter which intervention the study participant was allocated to. The meal consisted of white wheat bread, a pastry, butter, jam, cheese and 200 mL water (∼3000 kJ, 52.6 E% fat, 39.7 E% carbohydrate, 7.8 E% protein) and a standardised drink containing lactulose (5 g) and mannitol (2 g). Participants rated their well-being twice at fasting, and every 30 min following the standardised breakfast using a 100 mm visual analogue scale (VAS) with the most positive and the most negative ratings at each end of the line.

### Biochemical analyses of postprandial blood samples

Upon the standardised meal test, plasma glucose and serum insulin were measured in all postprandial blood samples as described above and plasma PYY, plasma GIP and plasma GLP-2 were measured in all postprandial blood samples as specified in Supplementary Methods.

### Exhalation of hydrogen

Hydrogen exhalation was measured twice at fasting, and every 30 min following the standardised breakfast and drink (*t* = 30, 60, 90, 120, 150 and 180 min). Breath hydrogen was measured in exhaled breath as a proxy measure of colonic fermentation using a handheld calibrated Gastro+Gastrolyzer^®^ (Bedfont Scientific Ltd.). Participants were instructed to breath in deeply; hold their breath for 15 s and then exhale at a steady pace into the cardboard mouthpiece of the device until their lungs felt empty.

### Visual analogue scoring of gastrointestinal indicators

Participants rated their well-being and gastrointestinal symptoms (bloating) during the past week using visual analogue scoring. The reliability and validity have been examined and a VAS score is considered to be a methodologically reliable measure of gastrointestinal comfort/discomfort^74^. Furthermore, participants provided information on smoking, intake of medications and dietary supplements and assessed their stool consistence on a 7-point scale (Bristol stool form scale) as well as their defaecation frequency^75^.

### Intestinal transit time

For six consecutive days before examination days 1, 2 and 4, the participants ingested 24 non-absorbable radio-opaque transit plastic ring markers in the morning on a daily basis to ensure saturation and filled in a defecation diary. Abdominal radiographs were performed in the afternoon at Frederiksberg Hospital, Copenhagen, Denmark on day 7 (examination day: the same day when faecal, blood and urine samples were collected), ∼30 h after the last transit marker intake. Intestinal transit time was estimated (ranked) based on the number of visible plastic markers on the obtained abdominal X-ray, adjusted for time since last marker ingestion. This was calculated as follows: number of markers counted from the X-ray film × the number of h between last marker ingestion and radiograph divided by 24 (daily dose of markers). The resulting relative transit time estimates enabled us to rank the participants according to transit time as reported^22^.

### Faecal calprotectin

Faecal calprotectin is used as a marker of inflammation of the small intestine, large bowel or the stomach. Calprotectin content in stools was measured using CALPROLAB^TM^ Calprotectin ELISA (ALP) (Calpro AS, Oslo, Norway), which is an ELISA based on polyclonal antibodies to human calprotectin with a reported CV% of 6.1–8.7% (S100A8/A9).

### Gut permeability assessment

After ingestion of the standardised breakfast and drink containing lactulose (5 g) and mannitol (2 g), urine was collected for 4 h. During the time of collection, urine was stored in the fridge. After collection, the urine samples were aliquoted into 2.0 mL tubes and stored at −80 °C. Quantification of lactulose in urine samples was performed by chromatographic analysis. Briefly, high-performance anion-exchange chromatography was performed with a Dionex CarboPac MA1 BioLC Analytical 4×250 mm column. The carbohydrate separation was performed using a Dionex CarboPac MA1 BioLC Guard 4×50 mm column (Dionex Corp, Sunnyvale, CA, USA). The samples were eluted with 50 mM NaOH at a flow rate of 1 mL min^−1^. Urinary excretion of mannitol was quantified using spectrophotometric analysis on a ABX Pentra 400 (Horiba Medical, California, USA). The CV% was 12.8 for lactulose and 0.8 for mannitol. The percentage of excreted lactulose and mannitol in urine after administration of the liquid formulation was evaluated, and the lactulose/mannitol ratio was calculated for each sample.

### Collection of urine samples

Upon arrival on each examination day, participants emptied their bladder. Urine was collected for 4 h after the standardised breakfast (containing approximately 3000 kJ, 52.6 E% fat, 39.7 E% carbohydrates, and 7–8 E% protein) and the lactulose-mannitol containing drink. Urine was stored in the fridge during collection, pooled, mixed and aliquoted into 2.0 mL tubes and stored at −80 °C. A complete set of the 4 h urine samples was available from 51 of the completing participants.

### Urine creatinine measurement

Creatinine concentrations were measured using urinary creatinine ELISA kit from Arbor Assays (Ann Arbor, Michigan, USA). All samples were diluted 1:20 and measured in duplicates (CV% was 1.7%). The range of the creatinine standard curve was 0.31–20 mg dL^−1^. Creatinine concentrations were used to adjust the injection volume of each urine sample when analysed by UPLC-MS as well as to normalise GC-MS data to account for the dilution of urine.

### Metabolomics

Untargeted urine metabolomics as well as targeted serum and faeces metabolomics quantifying short-chain fatty acids, fatty acids, acyl-carnitines, BAIBA and metabolites associated with the microbiota-gut-brain axis including serotonin, kynurenine, glutamate, γ-aminobutyric acid were performed by UPLC-MS and GC-MS. Details are available in Supplementary Methods.

### Statistical analyses

All statistical analyses were performed in R version 3.1 (The R Foundation for Statistical Computing, 2012, Vienna, Austria)^76^. Available-case analyses were carried out for all outcomes. The effects of the interventions on all outcomes were analysed by a linear mixed model (LMM) using the lme4 R-package^77^ with participant-specific and within-period participant-specific random effects. The model included an intervention–visit interaction and adjustment for age and gender as fixed effects. In addition, adjustment for intestinal transit time was included since this parameter recently has been reported to be an important confounder^22^. The effects of the intervention were assessed using the multcomp R-package^78^. Four individuals underwent antibiotics treatment during the trial and visits following antibiotics treatment were excluded from all statistics. Further details on statistical analyses are available in Supplementary Methods.

## Electronic supplementary material

Supplementary Information

Description of Additional Supplementary Files

Supplementary Data 1

Supplementary Data 2

Supplementary Data 3

Supplementary Data 4

Supplementary Data 5

Supplementary Data 6

Reporting Summary

## Data Availability

The raw Illumina read data for all samples have been deposited in the Short Read Archive under the Bioproject: PRJNA491335. Other data supporting the findings of the study are available in this article and its Supplementary Information files, or from the corresponding authors upon request.
