# Supplementary material for: A low-gluten diet induces changes in the intestinal microbiome of healthy Danish adults
Source: Nat Commun. 2018 Nov 13;9:4630. doi: 10.1038/s41467-018-07019-x (PMC6234216; doi:10.1038/s41467-018-07019-x)
Supplement: Supplementary file 1 — Supplementary Information [file 41467_2018_7019_MOESM1_ESM.pdf]

SUPPLEMENTARY MATERIALS

A low-gluten diet induces changes in the intestinal microbiome of healthy Danish adults

Hansen, Roager, Søndertoft, Gøbel *et al*.

Contains:

Supplementary Table 1-13
Supplementary Figure 1-8

Supplementary Methods
- Biochemical analyses of postprandial blood samples
- Dietary fibre composition of the two diets
- Resistant starch composition of the dietary study products
- FODMAP composition of the dietary study products
- Metagenomic sequencing
- Metagenomics analyses
- Quantitative PCR of *Bifidobacterium* spp. in faecal samples
- Urine metabolomics by UPLC-MS
- Urine metabolomics by GC-MS
- Serum and faeces metabolomics
- SCFA in plasma and faeces by LC-MS
- Statistical analyses

Supplementary References

SUPPLEMENTARY TABLES

Supplementary Table 1 Summary statistics of physiological traits for the 54 participants with more than one visit.

	All (n=54)	Men (n=23)	Women (n=31)	Control in period 1 (n=25; 13 men; 12 women)	Control in period 2 (n=29; 10 men; 19 women)
Age (y)	49,1 (11,2)	51,3 (9,8)	47,5 (12,0)	50,7 (9,1)	47,7 (12,7)
Body Mass Index (kg m^{-2})	28,7 (3,5)	29,3 (2,3)	28,3 (4,1)	28,5 (2,6)	28,9 (4,2)
Systolic blood pressure (mmHg)	128,0 (13,3)	135,2 (11,9)	122,7 (11,9)	128,8 (12,4)	127,3 (14,3)
Diastolic blood pressure (mmHg)	81,4 (9,4)	84,3 (8,6)	79,4 (9,6)	81,3 (10,1)	81,6 (8,7)
Smoker (%)	13.0	8.7	16.1	10.3	16
**Biochemistry in the fasting state**					
Plasma glucose (mmol L^{-1})	5,7 (0,7)	5,9 (0,6)	5,6 (0,7)	5,9 (0,7)	5,6 (0,7)
Serum triglycerides (mmol L^{-1})*	1,1 (0,7)	1,6 (1,3)	1,1 (0,4)	1,1 (0,7)	1,2 (0,9)
Serum HDL cholesterol (mmol L^{-1})	1,3 (0,3)	1,1 (0,3)	1,4 (0,3)	1,2 (0,3)	1,3 (0,3)
Whole-blood hemoglobin (mmol L^{-1})	8,4 (0,7)	9,0 (0,4)	8,0 (0,5)	8,6 (0,7)	8,3 (0,7)

Data are mean (SD). For non-normally distributed variables median (range) values are reported (*). Systolic and diastolic blood pressure is reported as a mean of two measurements. HDL: High-density lipoprotein.

Supplementary Table 2 Overview of macronutrient content of dietary study products, gluten and resistant starch composition in the low-gluten and high-gluten period

	Macronutrients[1]						Gluten[2]					Resistant starch[3]		
**Products (Brand)**	**Energy (kJ per 100 g)**	**Protein (g per 100 g)**	**Carbohydrate (g per 100 g)**	**Sugar (g per 100 g)**	**Fat (g per 100 g)**	**Fiber (g per 100 g)**	**Oat gluten (mg per 100 g)**	**Wheat gluten (mg per 100 g)**	**Rye gluten (mg per 100 g)**	**Barley gluten (mg per 100 g)**	**Total gluten (mg per 100 g)**	**RAS%**	**SDS%**	**RS%**
**Low-gluten products**														
Cornflakes (X-tra Coop)	1600	8	84	10	1	3.5	0	0	0	25.67	25.67	42±6	14±1	5.0±0.2
Oat flakes (Havreflakes AXA)	1650	12	68	12	6	9	9600	0	0	0	9600	29±2	4±0.1	3.3±0.04
Gluten-free pea fiber rye bread (Specialbageren)	891	3	40	2.5	3.6	6.7						40±2	5±0.1	2.2±0.1
Gluten-free bun (Homemade- NEXS kitchen)	1083	3.4	45.2	1.1	7.6	2.6						37±1	2±0.5	1.2±0.2
Gluten-free crispbread (WASA)	1600	5.5	72	2.5	7	6						31±2	36±1	4.8±0.7
White rice (First Price)	1550	8	80	-	1	1						23±3	29±1	0.50±0.01
Gluten-free pasta (Doves Farm)	1385	7.9	70.3	0.5	1.5	4.1						30±3	20±2	0.70±0.03
Quinoa (Urtekram)	1490	14	57	5	6	7						20±1	10±1	0.06±0.01
**High-gluten products**														
Wheat-rice flakes (Kellogs)	1606	14	76	17	0.5	2.5	0	6128	0	5	6133	17±3	27±7	1.6±0.3
Oat flakes (Havreflakes AXA)	1650	12	68	12	6	9	9600	0	0	0	9600	29±2	4±0.1	3.3±0.04
Maslin rye bread (Klassisk Bondebrød Kohberg)	990	7	47	3	1	4.5	0	2184	2301	0.46	4485.46	22±1	30±2	1.2±0.7
Wheat buns (Kohberg)	1000	9	45	3.5	1.5	2.5	0	7200	0	0	7200	21±3	34±2	1.6±0.1
Crisp bread (Frukost WASA)	1750	12	66	4	10	5	0	8808	0	0	8808	21±3	20±1	5.0±1.0
Bulgur (UNIFOOD)	1344	12	63	0	2	?	0	9600	0	0	9600	14±3	20±0.2	0.40±0.02
Wheat pasta (Spagetti Kungsörnen)	1500	12	71	2.5	2	3.5	0	9600	0	0	9600	20±2	16±1	0.80±0.03
Pearled spelt kernels (Lantmännen)	1450	13	61	0.5	3	7.5	0	10400	0	0	10400	19±0.01	13±2	2.0±0.2

Abbreviations: RAS: Rapidly absorbable sugars, SDS: slowly digestible starch, RS: Resistant Starch (RS)
[1]Data is from nutrient declarations.
[2]Calculated from Frida Food Data (Danish food database)[1]
[3]Experimental values - numbers are given in % dry matter (SD).

Supplementary Table 3 Consumption of dietary study products per week during the 8-weeks low-gluten and high-gluten periods (mean ± SD)

Low-gluten products (Brand)	Product intake (g per week)	Product intake (%)	High-gluten products (Brand)	Product intake (g per week)	Product intake (%)
Cornflakes (Coop)	149 ± 86	7%	Wheat-rice flakes (Kellogs)	206 ± 113	11%
Oat flakes (AXA)	60 ± 52	3%	Oat flakes (AXA)	60 ± 49	3%
Gluten-free pea fiber rye bread (Specialbageren)	562 ± 224	28%	Maslin rye bread (Kohberg)	538 ± 204	30%
Gluten-free bun (Homemade- NEXS kitchen)	745 ± 253	37%	Wheat buns (Kohberg)	529 ± 225	29%
Gluten-free crispbread (WASA)	111 ± 54	6%	Crisp bread (WASA)	104 ± 64	6%
White rice (First Price)	152 ± 87	8%	Bulgur (UNIFOOD)	131 ± 81	7%
Gluten-free pasta (Doves Farm)	125 ± 62	6%	Wheat pasta (Kungsörnen)	148 ± 74	8%
Quinoa (Urtekram)	84 ± 62	4%	Pearled spelt kernels (Lantmännen)	97 ± 53	5%
In total	1988 ± 539	100%	***In total***	1812 ± 467	100%

Data are given as consumption per week from 51 individuals completing both intervention arms and are presented as mean (SD).

Supplementary Table 4 Absolute plasma alkylresorcinol concentrations and changes hereof during the 8-weeks low-gluten and high-gluten interventions

	Low-gluten diet			High-gluten diet			
	**Week 0**	**Week 8**	**Change**	**Week 0**	**Week 8**	**Change**	**P value**
Total alkylresorcinol (nmol L^{-1})	139 (149)	13 (17)	-119 (147)	125 (122)	99 (90)	-27 (85)	<0,001

Data are from 51 individuals with complete data and are presented as median (IQR) (*). *P* value is derived from linear mixed models adjusted for changes in colonic transit time, serum triglycerides and low-density lipoprotein-cholesterol concentrations and study participant as a random variable (as justified by Lind *et al.*[2]).

Supplementary Table 5 Dietary intake of energy, macronutrients, certain food components and selected food groups in the low-gluten and in the high-gluten interventions

	Low-gluten period	High-gluten period	Mean of the difference	95% CI lower	95% CI upper	*P* value
**Energy and macronutrients**						
Energy total (kJ per day)	9574 (2868)	9559 (2310)	-15	-765	735	9.68E-01
Protein total (g per day)	78 (23)	85 (23)	7	2	12	1.13E-02
Fat total (g per day)	86 (37)	79 (28)	-7	-17	3	1.66E-01
Carbohydrate, total (g per day)	292 (85)	295 (76)	3	-19	25	7.96E-01
**Certain food components**						
Gluten (g per day)	2 (2)	18 (6)	16	15	18	2.20E-16
Wholegrain (g per day)	9 (9)	17 (13)	8	4	12	1.65E-04
Dietary fiber (g per day)	22 (7)	21 (7)	-1	-3	0	1.09E-01
**Food groups**						
Milk and milk products (g per day)	251 (207)	260 (181)	9	-31	49	6.45E-01
Cheese and cheese products (g per day)	34 (27)	34 (25)	-1	-9	8	9.01E-01
Cereals and starch products (g per day)	303 (85)	298 (84)	-5	-28	19	6.90E-01
Vegetables (g per day)	189 (135)	170 (109)	-11	-57	18	3.09E-01
Fruits (g per day)	182 (160)	158 (131)	-23	-59	13	2.10E-01
Meat (g per day)	99 (73)	106 (63)	6	-12	24	5.30E-01
Fish (g per day)	31 (26)	30 (22)	-2	-12	8	6.50E-01
Poultry (g per day) *	33 (40)	13 (29)	-1	-1	0	2.70E-02
Eggs (g per day) *	14 (23)	11 (26)	0	-1	0	4.53E-01
Potatoes (g per day) *	41 (45)	22 (45)	0	-1	0	3.18E-01
Beverages (g per day)	2154 (943)	2166 (724)	12	-156	180	8.87E-01

Data are from 50 individuals with complete diet data from both interventions arms and is presented as mean (SD) for normally distributed variables whereas median (range) is reported for non-normally distributed variables (*). Mean of difference and corresponding confidence interval and *P* value are derived from a paired t-test (high-gluten relative to low-gluten). Log transformation was applied to make data conform to normality when variables were non-normally distributed (*).

Supplementary Table 6 Change in *Bifidobacterium* abundance measured by qPCR during the low-gluten and high-gluten interventions

			Low-gluten vs. Baseline			High-gluten vs. Baseline			Low-gluten vs. High-gluten		
	Observations[1]	IDs[2]	Effect	Standard error	*P* value	Effect	Standard error	*P* value	Effect	Standard error	*P* value
Bifidobacterium abundance (*B. longum* DSM 20219 genomes per g faeces) Calculated with std curve.	187	49	-1.89	-6.50	7.80E-11	0.71	2.44	1.47E-02	-2.60	-6.37	1.90E-10
Total Bacterial load (16S copies per g faeces) Calculated with std curve.	187	49	-0.27	-1.64	1.00E-01	0.49	3.04	2.36E-03	-0.76	-3.33	8.64E-04
Bifidobacterium relative abundance - Calculated with std curves.	187	49	-1.63	-6.42	1.36E-10	0.21	0.81	4.16E-01	-1.84	-5.15	2.55E-07
Bifidobacterium relative abundance - Calculated with delta Ct method	187	49	-1.75	-6.40	1.50E-10	0.28	1.01	3.11E-01	-2.03	-5.28	1.26E-07

[1]Number of samples successfully analysed using qPCR after removing individuals with only one sample, samples missing intestinal transit time measure and samples taken immediately after antibiotic treatment.
[2]Number of successfully analysed individuals using qPCR after removing individuals with only one sample, samples missing intestinal transit time measure and samples taken immediately after antibiotic treatment.
The effect, standard error and P value of low-gluten vs. baseline and high-gluten vs. baseline are estimates of the mean change in the two periods derived from the LMM with an intervention-visit interaction adjusted for age, sex, intestinal transit time and with participant-specific, within-period participant-specific and qPCR plate batch number as random effects. The effect, standard error and P value of low-gluten vs. high-gluten are estimates of differences in change (high-gluten relative to low-gluten) and derived from a similar LMM. [1]Log transformation was applied to make data conform to normality.

Supplementary Table 7 Change in physiological measures during the low-gluten and high-gluten interventions

	Obser-vations*	IDs **	Low-gluten vs. Baseline Effect	Standard error	*P* value	High-gluten vs. Baseline Effect	Standard error	*P* value	Low-gluten vs. High-gluten Effect	Standard error	*P* value
**Anthropometrics**											
Body weight (kg)	204	54	-0.58	0.2	**1.11E-02**	0.23	0.2	3.21E-01	-0.81	0.3	**1.22E-02**
Waist circumference (cm) (mean of two)	204	54	-0.19	0.6	7.34E-01	-0.32	0.6	5.66E-01	0.14	0.8	8.61E-01
Saggital abdominal diamter (cm) (mean of two)	205	54	-0.54	0.2	**1.96E-03**	-0.16	0.2	3.81E-01	-0.38	0.2	1.19E-01
**Biochemistry of blood samples during fasting**											
Plasma glucose (mmol L^{-1})	205	54	0.00	0.1	9.41E-01	0.14	0.1	**2.43E-02**	-0.13	0.1	1.11E-01
Serum insulin (pmol L^{-1})[1]	198	54	-0.04	0.1	5.78E-01	0.02	0.1	8.11E-01	-0.05	0.1	5.62E-01
Serum C-peptide (pmol L^{-1})	202	54	-35.27	32.1	2.71E-01	14.08	32.2	6.62E-01	-49.35	43.9	2.61E-01
Homeostatic model assessment - insulin resistance (fasting insulin (iU mL^{-1}) x fasting glucose (mmol L^{-1}) x 22,5^{-1})*	198	54	-0.03	0.1	6.15E-01	0.03	0.1	6.25E-01	-0.07	0.1	4.68E-01
Whole-blood glycated hemoglobin (µmol L^{-1})	205	54	-0.01	0.0	7.23E-01	-0.01	0.0	6.90E-01	0.00	0.0	9.68E-01
Plasma gastric inhibitory peptide (pmol L^{-1})	203	54	-0.49	0.5	2.80E-01	0.52	0.5	2.54E-01	-1.02	0.6	9.92E-02
Serum triglycerides (mmol L^{-1})[1]	204	54	0.03	0.0	5.07E-01	-0.05	0.0	2.80E-01	0.08	0.1	2.05E-01
Serum total cholesterol (mmol L^{-1})	204	54	-0.20	0.1	**1.80E-02**	-0.10	0.1	2.54E-01	-0.10	0.1	3.87E-01
Serum HDL cholesterol (mmol L^{-1})	204	54	-0.02	0.0	3.27E-01	0.01	0.0	6.59E-01	-0.03	0.0	3.07E-01
Serum LDL cholesterol (mmol L^{-1})	204	54	-0.14	0.1	**4.96E-02**	-0.05	0.1	4.76E-01	-0.09	0.1	3.71E-01
Serum alanin-aminotransferase (U L^{-1})[1]	204	54	-0.05	0.1	3.12E-01	-0.04	0.1	4.39E-01	-0.01	0.1	8.73E-01
Serum aspartate aminotransferase (U L^{-1})[1]	204	54	-0.04	0.0	3.85E-01	-0.02	0.0	7.44E-01	-0.02	0.1	7.01E-01
Whole-blood hemoglobin (mmol L^{-1})	204	54	0.02	0.0	6.30E-01	-0.07	0.1	1.95E-01	0.09	0.1	1.95E-01
Whole-blood white blood cells (x10^9 L^{-1})	204	54	-0.18	0.1	1.62E-01	-0.03	0.1	8.10E-01	-0.15	0.2	4.03E-01
Whole-blood lymphocytes (x10^9 L^{-1})	205	54	-0.04	0.0	3.51E-01	0.06	0.0	1.71E-01	-0.10	0.1	9.38E-02
Mix of whole blood monocytes, mast cells, basophils, eosinophils (x10^9 L^{-1})	205	54	-0.01	0.0	6.56E-01	-0.02	0.0	3.57E-01	0.01	0.0	7.19E-01
Whole-blood neutrophils (x10^9 L^{-1})	205	54	-0.11	0.1	2.90E-01	-0.06	0.1	5.59E-01	-0.05	0.1	7.38E-01
Serum CRP (mg L^{-1})	200	52	-0.25	0.1	5.25E-02	0.08	0.1	5.66E-01	-0.33	0.2	6.64E-02
Serum IL-6 (pg mL^{-1})[1]	201	54	-0.11	0.1	1.71E-01	0.01	0.1	9.08E-01	-0.12	0.1	2.85E-01
Serum TNF-α (pg mL^{-1})[1]	202	54	-0.01	0.0	8.07E-01	0.00	0.0	9.65E-01	-0.01	0.0	8.37E-01
Serum zonulin (ng mL^{-1})	204	54	-0.22	0.1	**3.47E-02**	-0.09	0.1	3.69E-01	-0.12	0.1	3.87E-01
Plasma citrullin (µmol L^{-1})	203	54	-0.59	0.7	4.21E-01	-0.20	0.7	7.92E-01	-0.40	1.0	6.97E-01
**Postprandial measures**											
**Plasma glucose (mmol L^{-1})**											
Plasma glucose (mmol/L) (0 min)	205	54	0.00	0.1	9.41E-01	0.14	0.1	**2.43E-02**	-0.13	0.1	1.11E-01
Plasma glucose (mmol/L) (30 min)	205	54	0.09	0.1	3.77E-01	-0.06	0.1	5.28E-01	0.15	0.1	2.72E-01
Plasma glucose (mmol/L) (60 min)	203	54	0.03	0.1	8.47E-01	0.16	0.1	2.54E-01	-0.14	0.2	4.83E-01

Plasma glucose (mmol/L) (120 min)	205	54	-0.08	0.2	6.21E-01	-0.02	0.2	8.98E-01	-0.06	0.2	7.92E-01
Plasma glucose (mmol/L) (180 min)	202	54	0.02	0.1	8.31E-01	0.06	0.1	5.99E-01	-0.04	0.2	8.12E-01
Plasma glucose (mmol/L) (AUC)	200	54	0.38	16.5	9.81E-01	9.02	17.1	5.99E-01	-8.63	23.3	7.11E-01
Plasma glucose (mmol/L) (Global test[3])	1020	54	NA	NA	8.75E-01	NA	NA	5.76E-01	NA	NA	8.30E-01
**Serum insulin (pmol L^{-1}) cleaned for hemolysis**											
Plasma insulin (pmol/L) cleaned for hemolysis (0 min)[1]	198	54	-0.04	0.1	5.78E-01	0.02	0.1	8.11E-01	-0.05	0.1	5.62E-01
Plasma insulin (pmol/L) cleaned for hemolysis (30 min)[1]	203	54	-0.10	0.1	1.12E-01	-0.11	0.1	9.43E-02	0.01	0.1	9.38E-01
Plasma insulin (pmol/L) cleaned for hemolysis (60 min)[1]	198	54	0.05	0.1	5.08E-01	0.04	0.1	6.23E-01	0.01	0.1	9.03E-01
Plasma insulin (pmol/L) cleaned for hemolysis (120 min)[1]	199	54	-0.06	0.1	4.11E-01	-0.11	0.1	1.74E-01	0.04	0.1	6.84E-01
Plasma insulin (pmol/L) cleaned for hemolysis (180 min)[1]	193	54	-0.01	0.1	9.15E-01	0.01	0.1	9.27E-01	-0.01	0.1	8.85E-01
Plasma insulin (pmol/L) cleaned for hemolysis (AUC)	180	54	-0.01	0.0	7.66E-01	-0.03	0.1	5.84E-01	0.01	0.1	8.50E-01
Plasma insulin (pmol/L) cleaned for hemolysis (Global test[3])[1]	991	54	NA	NA	5.48E-01	NA	NA	3.16E-01	NA	NA	9.82E-01
**Plasma glucagon-like peptide 2**											
Plasma glucagon-like peptide 2 (0 min)	203	53	-0.53	1.1	6.47E-01	-0.67	1.2	5.65E-01	0.14	1.5	9.25E-01
Plasma glucagon-like peptide 2 (30 min)	203	53	-2.44	1.3	6.59E-02	-3.30	1.4	**1.46E-02**	0.86	1.8	6.36E-01
Plasma glucagon-like peptide 2 (60 min)	202	53	4.75	1.7	**4.57E-03**	-0.05	1.7	9.78E-01	4.79	2.3	**3.54E-02**
Plasma glucagon-like peptide 2 (120 min)	203	53	0.67	2.0	7.43E-01	-0.61	2.1	7.67E-01	1.28	2.8	6.42E-01
Plasma glucagon-like peptide 2 (180 min)	198	53	0.82	1.5	5.89E-01	1.48	1.6	3.49E-01	-0.65	2.1	7.55E-01
Plasma glucagon-like peptide 2 (AUC)	197	53	192.44	213.2	3.67E-01	-73.17	223.3	7.43E-01	265.60	295.6	3.69E-01
Plasma glucagon-like peptide 2 (Global test[3])	1009	53	NA	NA	**4.35E-02**	NA	NA	4.37E-01	NA	NA	2.22E-01
**Plasma gastric inhibitory peptide (pM)**											
Plasma gastric inhibitory peptide (pM) (0 min)	203	54	-0.49	0.5	2.80E-01	0.52	0.5	2.54E-01	-1.02	0.6	9.92E-02
Plasma gastric inhibitory peptide (pM) (30 min)	203	54	-3.80	3.8	3.16E-01	-5.67	4.0	1.51E-01	1.87	5.3	7.26E-01
Plasma gastric inhibitory peptide (pM) (60 min)	201	54	-6.67	5.6	2.33E-01	-4.40	5.8	4.52E-01	-2.28	7.8	7.71E-01
Plasma gastric inhibitory peptide (pM) (120 min)	199	54	-3.70	6.2	5.48E-01	-14.09	6.2	**2.35E-02**	10.39	8.3	2.13E-01
Plasma gastric inhibitory peptide (pM) (180 min)	192	54	-0.19	3.6	9.57E-01	1.13	3.7	7.59E-01	-1.32	4.9	7.89E-01
Plasma gastric inhibitory peptide (pM) (AUC)	184	54	-648.94	559.2	2.46E-01	-891.94	590.3	1.31E-01	243.00	789.9	7.58E-01
Plasma gastric inhibitory peptide (pM) (Global test[3])	998	54	NA	NA	6.51E-01	NA	NA	**1.20E-02**	NA	NA	4.82E-01
**Plasma peptide-YY (pM)**											
Plasma peptide-YY (pM) (0 min)	203	54	-0.07	0.5	8.96E-01	-0.56	0.5	2.75E-01	0.49	0.7	4.77E-01
Plasma peptide-YY (pM) (30 min)	204	54	0.61	0.6	2.71E-01	-0.85	0.6	1.32E-01	1.46	0.8	5.77E-02
Plasma peptide-YY (pM) (60 min)	200	54	1.45	0.7	**2.92E-02**	-0.97	0.7	1.56E-01	2.42	0.9	**9.13E-03**
Plasma peptide-YY (pM) (120 min)	203	54	-0.10	0.7	8.84E-01	-1.97	0.7	**7.01E-03**	1.87	1.0	6.05E-02
Plasma peptide-YY (pM) (180 min)	199	54	-0.17	0.6	7.84E-01	-0.68	0.7	2.96E-01	0.51	0.9	5.65E-01

Plasma peptide-YY (pM) (AUC)	194	54	65.34	84.1	4.37E-01	-234.43	88.0	**7.75E-03**	299.77	119.0	**1.18E-02**
Plasma peptide-YY (pM) (Global test***)	1009	54	NA	NA	3.22E-01	NA	NA	**1.51E-02**	NA	NA	**9.70E-03**
**Plasma free fatty acids (mmol L^{-1})**											
Plasma free fatty acids (mmol L^{-1}) (0 min)	204	54	0.02	0.0	4.68E-01	0.01	0.0	6.17E-01	0.01	0.0	8.71E-01
Plasma free fatty acids (mmol L^{-1}) (30 min)	205	54	0.03	0.0	1.20E-01	0.00	0.0	9.03E-01	0.03	0.0	2.92E-01
Plasma free fatty acids (mmol L^{-1}) (60 min)	203	54	0.02	0.0	6.32E-02	0.01	0.0	4.14E-01	0.01	0.0	4.59E-01
Plasma free fatty acids (mmol L^{-1}) (120 min)	202	54	0.00	0.0	6.23E-01	0.01	0.0	2.47E-01	-0.02	0.0	2.19E-01
Plasma free fatty acids (mmol L^{-1}) (180 min)	196	54	0.00	0.0	7.81E-01	0.00	0.0	9.02E-01	0.00	0.0	7.68E-01
Plasma free fatty acids (mmol L^{-1}) (AUC)	194	54	1.81	2.0	3.64E-01	1.54	2.1	4.58E-01	0.28	2.7	9.19E-01
Plasma free fatty acids (mmol L^{-1}) (Global test[3])	1010	54	NA	NA	1.30E-01	NA	NA	9.93E-01	NA	NA	4.62E-01
**Hydrogen exhalation (ppm)**											
Exhalation of H2 (ppm) (baseline)1	205	54	-0.57	0.1	**4.59E-07**	-0.17	0.1	1.47E-01	-0.40	0.2	**9.42E-03**
Exhalation of H2 (ppm) (0 min)[1]	205	54	-0.51	0.1	**7.88E-05**	-0.08	0.1	5.44E-01	-0.43	0.2	**1.43E-02**
Exhalation of H2 (ppm) (30 min)[1]	204	54	-0.51	0.1	**3.50E-05**	-0.04	0.1	7.29E-01	-0.47	0.2	**5.87E-03**
Exhalation of H2 (ppm) (60 min)[1]	205	54	-0.44	0.1	**3.91E-04**	-0.07	0.1	5.76E-01	-0.37	0.2	**3.06E-02**
Exhalation of H2 (ppm) (90 min)[1]	204	54	-0.39	0.1	**1.90E-04**	-0.02	0.1	8.16E-01	-0.36	0.1	**1.18E-02**
Exhalation of H2 (ppm) (120 min)[1]	204	54	-0.29	0.1	**3.71E-03**	-0.09	0.1	3.63E-01	-0.20	0.1	1.51E-01
Exhalation of H2 (ppm) (150 min)[1]	205	54	-0.26	0.1	**6.41E-03**	-0.14	0.1	1.52E-01	-0.12	0.1	3.63E-01
Exhalation of H2 (ppm) (180 min)[1]	205	54	-0.18	0.1	**5.25E-02**	-0.08	0.1	3.98E-01	-0.10	0.1	4.37E-01
Exhalation of H2 (ppm) (AUC)[1]	202	54	-0.46	0.1	**4.43E-06**	-0.10	0.1	3.17E-01	-0.35	0.1	**1.00E-02**
Exhalation of H2 (ppm) (Global test[3])	1605	54	NA	NA	**2.78E-06**	NA	NA	6.38E-01	NA	NA	**2.12E-08**
**Subjective measures**											
Overall well-being (mm)	1806	54	NA	NA	6.39E-01	NA	NA	**1.72E-04**	NA	NA	**1.33E-06**
Bloating (mm)[2]	205	54	-1.07	0.5	**3.63E-02**	1.52	0.6	**8.90E-03**	-2.59	0.8	**8.87E-04**
Gas or flatulence (mm)[2]	204	54	-0.59	0.5	2.72E-01	0.97	0.7	1.49E-01	-1.56	0.8	6.38E-02
Average number of defaecations over the last week (number)	204	54	-0.07	0.1	3.34E-01	-0.09	0.1	2.30E-01	0.02	0.1	8.50E-01
Bristol Stool Scale	189	54	-0.14	0.2	4.83E-01	0.04	0.2	8.55E-01	-0.18	0.3	5.16E-01
**Markers of gastrointestinal health**											
Intestinal Transit Time (day)	206	54	0.13	0.1	2.00E-01	0.20	0.1	**4.54E-02**	-0.07	0.1	5.90E-01
Fecal calprotectin (mg per kg faeces)[2]	196	53	0.00	0.5	9.94E-01	0.15	0.5	7.72E-01	-0.16	0.7	8.26E-01
Urine lactulose-mannitol ratio	203	54	0.004	0.003	1.25E-01	0.005	0.003	**4.23E-02**	-0.001	0.004	6.83E-01
**Ex vivo cytokine production[4]**											
Ex vivo IL-1β production from 50 µL whole blood after 24h stimulation with 0,1 µg mL^{-1} of LPS, (pg mL^{-1})	206	54	-212.1	187.1	2.57E-01	330.73	193.4	8.73E-02	-542.84	257.1	**3.48E-02**
Ex vivo TNF-α production from 50 µL whole blood after 24h stimulation with 0,1 µg mL^{-1} of LPS, (pg mL^{-1})[1]	206	54	-0.30	0.1	**7.27E-03**	-0.14	0.1	2.09E-01	-0.15	0.2	3.12E-01
Ex vivo IL-6 production from 50 µL whole blood after 24h stimulation with 0,1 µg mL^{-1} of LPS, (pg mL^{-1})	207	54	-3553.3	993.7	**3.49E-04**	-1068.2	1020.0	2.95E-01	-2485.1	1358.1	6.73E-02

Ex vivo IFN-γ production from 50 µL whole blood after 24h stimulation with 0,1 µg mL^{-1} of LPS, (pg mL^{-1})[1]	170	54	0.02	0.3	9.56E-01	0.22	0.3	4.13E-01	-0.20	0.4	5.95E-01

* Number of successful observations out of a total of 205 observation possible after removing individuals with only one sample and samples missing CTT measure. For outcomes with five measure at visit, the total number of observations possible is 1025 and eight measures is 1640. Overall well-being was measured 9 times after a standardised meal and the total number of possible observations is 1845. CTT and ex vivo cytokine production measures were not corrected for CTT and the total number of possible observations is 212.

** Number of individuals out of a total of 54 individuals possible after removing individuals with only one sample, samples missing intestinal transit time measure and samples taken immediately after antibiotic treatment.
The effect, standard error and P value of low-gluten vs. baseline and high-gluten vs. baseline are estimates of the mean change in the two periods derived from the LMM with an intervention-visit interaction adjusted for age, sex, intestinal transit time and with participant-specific and within-period participant-specific specific random effects. The effect, standard error and P value of low-gluten vs. high-gluten are estimates of differences in change (high-gluten relative to low-gluten) and derived from a similar LMM. For outcomes measured at more than one time point the intervention-visit interaction was replaced with a three-way treatment-visit-time interaction and the global effect was evaluated with a chi square test.
[1]Log transformation was applied to make data conform to normality when variables were non-normally distributed, [2]if data remained non-normal these were dichotomized. This applied for gastrointestinal comfort indicators for which 1 was >5 mm and 0 was =< 5 mm. [3]Global tests were evaluated with a chi square test. [4]Ex vivo immunity data are a mean of three replicates from 54 individuals completing >2 visits. Waist circumference and sagittal abdominal diameter are reported as means of two measurements. Homeostatic model assessment - insulin resistance (HOMA-IR) is calculated as fasting insulin (iU mL^{-1}) x fasting glucose (mmol L^{-1}) x 22,5^{-1}). The values for colonic transit time from visit 1 and 3 are assumed to be similar. Area under the curve (AUC) is calculated using all time points. AUC: area under the curve, CRP: C-reactive protein; HDL: high-density lipoprotein; HOMA-IR: homeostatic model assessment - insulin resistance, IFN-γ: interferon gamma; IL-: interleukin-; LDL: low-density lipoprotein; LPS: lipopolysaccharides; TNF-α: tumor necrosis factor alpha.

Supplementary Table 8 Targeted associations between significant manually curated reference modules and hydrogen exhalation, significant ex vivo cytokine responses and urine concentration of β-aminoisobutyric acid

	Variable 1	Module description	Variable 2	Effect	*P* value
**Gas metabolism**	MF0069	NADH:ferredoxin oxidoreductase	Exhalation of H2 (AUC)	0.00E+00	9.69E-01
	MF0073	Pyruvate:ferredoxin oxidoreductase	Exhalation of H2 (AUC)	-2.00E-04	3.11E-01
	MF0081	Methanol conversion	Exhalation of H2 (AUC)	-6.90E-03	**2.10E-02**
	MF0097	Homoacetogenesis	Exhalation of H2 (AUC)	-6.00E-04	3.97E-01
	MF0098	Hydrogen metabolism	Exhalation of H2 (AUC)	-4.00E-04	7.70E-02
	MF0099	Methanogenesis - methyl-coM	Exhalation of H2 (AUC)	-9.20E-03	**4.00E-03**
	MF0100	Methanogenesis from carbon dioxide	Exhalation of H2 (AUC)	-9.70E-03	**5.00E-03**
	MF0102	Sulfate reduction (dissimilatory)	Exhalation of H2 (AUC)	1.00E-03	1.92E-01
**LPS Synthesis**	M00060	Lipopolysaccharide biosynthesis, KDO2-lipid A	LPS_IL1b_mean	1.60E-04	**4.26E-02**
	M00060	Lipopolysaccharide biosynthesis, KDO2-lipid A	LPS_IL6_mean	2.71E-05	1.32E-01
**BAIBA**	M00046	Pyrimidine degradation	β-aminoisobutyric acid	1.18E-01	1.83E-01
	M00046	Pyrimidine degradation	mz104.070707401539	1.43E-01	2.21E-01
**SCFA**	MF0086	Acetyl-CoA to acetate	Plasma peptide-YY	1.66E-05	9.46E-01
	MF0087	Acetyl-CoA to crotonyl-CoA	Plasma peptide-YY	-4.44E-04	5.81E-01
	MF0088	Butyrate production I	Plasma peptide-YY	-1.54E-04	8.70E-01
	MF0089	Butyrate production II	Plasma peptide-YY	-5.26E-04	4.67E-01
	MF0093	Propionate production I	Plasma peptide-YY	-3.87E-03	2.96E-01
	MF0094	Propionate production II	Plasma peptide-YY	2.47E-04	8.25E-01
	MF0095	Propionate production III	Plasma peptide-YY	1.32E-03	2.72E-01

The effect, standard error and *P* value are estimates of the correlations between mean changes in two variables using a LMM with customized reference modules as response and breath hydrogen, significant *ex vivo* cytokine production and β-aminoisobutyric acid (BAIBA) as a continuous variable when correcting for age, gender, intestinal transit time and individual as a random factor. LPS, lipopolysaccharides; SCFA, short-chain fatty acids.

Supplementary Table 9 List of urine metabolites differing between the low-gluten and high-gluten interventions identified by UPLC-MS

									Low-gluten vs High-gluten[b]					
**Tentative structural assignment**	**ID level[a]**	**Adduct**	**Retention time**	**Experimental *m/z***	**MS/MS fragmentation *m/z***	**Authentic standard *m/z***	**Database/ theoretical *m/z***	**Mass error**	**Observations**	**IDs**	**Effect size**	**Standard error**	***P* value**	**Adjusted *P* value[c]**
			min					*mDa*						
Unknown	4	$[M+H]^+$	0.5	179.0035	Not obtained		-	-	197	51	-0.1	0.03	4.50E-04	3.80E-02
Unknown	4	$[M+H]^+$	0.6	222.0451	Not obtained		-	-	197	51	0.11	0.03	4.50E-04	3.80E-02
Guanidinosuccinic acid	2	$[M+H]^+$	0.7	176.0667	158.0557 $[M+H-H_2O]^+$, 134.0449, 114.1129, 88.0397		176.0666	0.1	197	51	-0.07	0.02	1.50E-04	1.50E-02
β-aminoisobutyric acid (BAIBA)[d]	1	$[M+H]^+$	0.7	104.0707		104.0707 BAIBA [M+H]+	104.0706	0.1	197	51	0.12	0.04	4.70E-04	3.80E-02
Galactosylglycerol	2	$[M+H]^+$	0.7	255.1065	192.0324, 144.1020, 127.0501		255.1074	0.9	197	51	-0.22	0.04	2.00E-09	5.20E-07
N-Acetylcystathionine	2	$[M+H]^+$	0.8	265.0851	223.0756 $[M+H-acetyl]^+$ (cystathionine fragment), 110.0704		265.0853	0.2	197	51	0.28	0.06	4.90E-06	1.00E-03
Unknown	4	$[M+H]^+$	1.3	206.0116	Not obtained		-	-	197	51	0.08	0.02	5.70E-05	7.00E-03
2,3-Diaminosalicylic acid	2	$[M+H]^+$	2.2	169.0607	151.0503, 141,0663, 125.0714, 123.0555		169.0608	0.1	197	51	0.55	0.1	1.30E-07	2.40E-05
Unknown	4	$[M+H]^+$	2.8	197.0558	169.0610 $[M+H-CO]^+$, 153.0660, 135.0448, 123.0545		-	-	197	51	0.35	0.08	1.30E-05	2.00E-03
Phenol metabolite	3	$[M+H]^+$	3.2	271.1286	253.1181 $[M-H_2O+H]^+$, 192.1017, 165.1373, 148.1122, 120.0816, 84.0810		-	-	197	51	-0.2	0.04	1.60E-06	2.70E-04
		$[M-H_2O+H]^+$	3.2	253.1181	192.1016, 148.1120		-	-	197	51	-0.27	0.05	4.70E-09	1.00E-06
	1		3.2	376.1237		183.065	376.1238	0.1	197	51	-0.53	0.07		8.70E-13

3,5-dihydroxy-hydrocinnamic acid-glucuronide (DHPPA-glucuronide)		[M+NH$_4$]$^+$			183.0651 [M+H-glucuronide]$^+$, 165.0547 [M+H-glucuronide-H_2O]$^+$	3,5-dihydro-xyhydrocinnamic acid [M+H]$^+$							6.70E-16	
Unknown	4	[M+H]$^+$	4.5	226.1119	Not obtained		-	-	197	51	-0.08	0.02	9.70E-05	1.00E-02
Enterolactone-glucuronide	1	[M+NH$_4$]$^+$	5.4	492.1863	321.1104 [M+Na-glucuronide]$^+$, 299.1274 [M+H-glucuronide]$^+$, 281.1170 [M+H-glucuronide-H_2O]$^+$, 263.1070 [M+H-glucuronide-2H_2O]$^+$,185.1162	299.1278 Enterolactone [M+H]$^+$	492.1864	0.1	197	51	0.78	0.1	7.40E-14	4.80E-11
		[M+Na]$^+$	5.4	497.1417	321.1095 [M+Na-glucuronide]$^+$, 300.1312, 299.1272 [M+H-glucuronide]$^+$, 281.1161, 185.1179	299.1278 Enterolactone [M+H]$^+$	497.1418	0.1	197	51	0.69	0.1	1.20E-11	5.30E-09
		[M+H-glucuronide]$^+$	5.4	299.1277	281.1166 [M+H-H_2O]$^+$, 263.1064 [M+H-2H_2O]$^+$, 149.0240, 133.0644, 107.0496	299.1278 Enterolactone [M+H]$^+$	299.1278	0.1	197	51	0.71	0.11	1.30E-10	4.30E-08

m/z: mass-to-charge

[a]Identification levels by Metabolomics Standard Initiative[3].

[b]Overall intervention effect assessed by LMM adjusting for age, sex and transit time.

[c]q values are adjusted p-values by the Benjamini Hochberg approach[4].

[d]β-aminoisobutyric acid (BAIBA) was also found to be significantly different with GC-MS (q<0.0001)

Supplementary Table 10 - Associations between significantly changing urine metabolites and KEGG modules

Variable 1		Variable 2					
m/z	Annotation	Module	Annotation	Effect	Standard error	*P* value	Adjusted *P* value
197.0558	*m/z* 197.0558	M00342	Pentose phosphate pathway (non-oxidative branch)	-0.093	0.02	2.91E-06	1.62E-03
255.1065	Galactosylglycerol	M00233	PTS system, sucrose-specific II component	0.368	0.08	8.96E-06	1.66E-03
197.0558	*m/z* 197.0558	M00233	PTS system, sucrose-specific II component	-0.172	0.04	6.71E-06	1.66E-03
255.1065	Galactosylglycerol	M00244	PTS system, glucitol/sorbitol-specific II component	0.367	0.09	4.06E-05	5.64E-03
376.1236	DHPPA-glucuronide	M00290	Sucrose degradation I	0.196	0.05	8.54E-05	9.48E-03
376.1236	DHPPA-glucuronide	MF0011	Fatty acid biosynthesis, initiation	0.299	0.08	3.20E-04	2.96E-02
255.1065	Galactosylglycerol	M00342	Pentose phosphate pathway (non-oxidative branch)	0.154	0.04	3.85E-04	3.05E-02
104.0707	β-aminoisobutyric acid	MF0010	Nucleotide sugar biosynthesis, glucose=> UDP-glucose	-1.810	0.52	5.40E-04	3.33E-02
255.1065	Galactosylglycerol	M00281	Arabinose degradation	0.323	0.09	5.18E-04	3.33E-02
255.1065	Galactosylglycerol	M00269	PTS system, mannose-specific II component	0.641	0.19	9.80E-04	4.95E-02
169.0607	2,3-Diaminosalicylic acid	M00342	Pentose phosphate pathway (non-oxidative branch)	-0.057	0.02	9.79E-04	4.95E-02

The effect, standard error and *P* value are estimates of the correlations between mean changes in two variables using a LMM with modules as response and urine metabolites and breath hydrogen as a continuous variable when correcting for age, gender, intestinal transit time and individual as a random factor. *m/z*, mass-to-charge; DHPPA, 3,5-dihydroxy-hydrocinnamic acid-glucuronide

Supplementary Table 11 Change in faecal and serum short chain fatty acids (SCFA) during the low-gluten and high-gluten interventions

	Observa-tions[1]	IDs[2]	Low-gluten vs. Baseline			High-gluten vs. Baseline			Low-gluten vs. High-gluten		
			Effect	Standard error	*P* value	Effect	Standard error	*P* value	Effect	Standard error	*P* value
**FAECES**											
Acetate	197	53	-0.22	0.2	2.19E-01	0.07	0.2	6.94E-01	-0.29	0.2	2.51E-01
Propionate	197	53	-0.20	0.2	2.50E-01	0.09	0.2	6.11E-01	-0.30	0.3	2.40E-01
Butyrate	197	53	-0.26	0.2	1.49E-01	0.01	0.2	9.47E-01	-0.28	0.3	2.86E-01
2-methylbutyrate	197	53	-0.19	0.2	2.73E-01	0.16	0.2	3.53E-01	-0.35	0.2	1.51E-01
Isovalerate	197	53	-0.18	0.2	2.97E-01	0.16	0.2	3.57E-01	-0.33	0.2	1.64E-01
Valerate	197	53	-0.24	0.2	1.84E-01	0.07	0.2	6.98E-01	-0.31	0.3	2.23E-01
Caproate	197	53	-0.36	0.2	6.75E-02	-0.03	0.2	8.81E-01	-0.33	0.3	2.32E-01
Enanthate	197	53	-0.28	0.2	1.78E-01	0.00	0.2	9.87E-01	-0.29	0.3	3.31E-01
Isobutyrate	197	53	-0.24	0.2	1.71E-01	0.10	0.2	5.80E-01	-0.33	0.2	1.73E-01
Caprylate	197	53	-0.37	0.3	2.06E-01	0.01	0.3	9.71E-01	-0.38	0.4	3.55E-01
**SERUM**											
Acetate	194	53	0.16	0.1	6.18E-02	0.12	0.1	1.42E-01	0.03	0.1	7.79E-01
Propionate	194	53	0.05	0.1	5.37E-01	0.02	0.1	7.87E-01	0.03	0.1	8.06E-01
Butyrate	194	53	0.05	0.1	4.51E-01	0.06	0.1	2.84E-01	-0.02	0.1	8.21E-01
Isobutyrate	194	53	0.08	0.1	2.87E-01	0.06	0.1	4.07E-01	0.02	0.1	8.68E-01
Lactate	194	53	-0.02	0.0	5.41E-01	0.00	0.0	9.86E-01	-0.02	0.0	6.73E-01
2-methylbutyrate	194	53	0.04	0.1	5.55E-01	0.04	0.1	5.66E-01	0.00	0.1	9.91E-01
Isovalerate	194	53	-0.03	0.1	5.97E-01	0.05	0.1	3.81E-01	-0.09	0.1	3.18E-01
Valerate	194	53	0.01	0.1	9.01E-01	0.08	0.1	5.16E-01	-0.06	0.2	7.09E-01
3OH-butyrate	194	53	0.11	0.1	4.10E-01	0.06	0.1	6.44E-01	0.05	0.2	7.97E-01
Caproate	194	53	0.01	0.1	9.69E-01	0.06	0.1	6.51E-01	-0.05	0.2	7.69E-01
Enanthate	194	53	0.00	0.2	9.87E-01	-0.02	0.2	8.95E-01	0.02	0.2	9.35E-01
Caprylate	194	53	0.05	0.1	6.51E-01	0.01	0.1	9.24E-01	0.04	0.1	8.01E-01

[1]Number of samples successfully analysed after removing individuals with only one sample, samples missing intestinal transit time measure and samples taken immediately after antibiotic treatment.

[2]Number of successfully analysed after removing individuals with only one sample, samples missing intestinal transit time measure and samples taken immediately after antibiotic treatment.

The effect, standard error and P value of low-gluten vs. baseline and high-gluten vs. baseline are estimates of the mean change in the two periods derived from the LMM with an intervention-visit interaction adjusted for age, sex, intestinal transit time and with participant-specific and within-period participant-specific specific random effects. The effect, standard error and P value of low-gluten vs. high-gluten are estimates of differences in change (high-gluten relative to low-gluten) and derived from a similar LMM. Log transformation was applied to make data conform to normality.

Supplementary Table 12 Change in targeted faeces and serum metabolites related to the gut-brain axis and lipid metabolism during the low-gluten and high-gluten interventions

				Low-gluten vs. Baseline			High-gluten vs. Baseline			Low-gluten vs. High-gluten		
	Platform	Observa-tions[1]	IDs[2]	Effect	Standard error	*P* value	Effect	Standard error	*P* value	Effect	Standard error	*P* value
**FAECES**												
**Aromatic amino acids and derivatives**												
Tyrosine	LC-MS	195	53	-0.19	0.2	3.16E-01	0.08	0.2	6.94E-01	-0.27	0.3	3.22E-01
Tryptophan	LC-MS	195	53	-0.11	0.2	5.63E-01	0.11	0.2	5.49E-01	-0.22	0.3	4.04E-01
5-hydroxyindoleacetic acid	GC-MS	194	53	0.59	0.4	1.79E-01	-0.11	0.4	8.04E-01	0.70	0.6	2.54E-01
4-hydroxymandelic acid	GC-MS	194	53	0.16	0.4	6.87E-01	-0.20	0.4	6.18E-01	0.36	0.6	5.22E-01
Indoleacetic acid	LC-MS	195	53	-0.12	0.3	6.36E-01	-0.11	0.3	6.78E-01	-0.02	0.4	9.66E-01
**Gut-brain**												
Kynurenine	LC-MS	195	53	1.00	0.3	**3.63E-03**	-0.34	0.3	3.19E-01	1.35	0.5	**5.49E-03**
Serotonin	LC-MS	195	53	-0.21	0.3	4.41E-01	-0.13	0.3	6.38E-01	-0.08	0.4	8.30E-01
Glutamic acid	LC-MS	195	53	-0.23	0.2	2.51E-01	0.09	0.2	6.66E-01	-0.32	0.3	2.61E-01
Gamma-aminobutyric acid (GABA)	GC-MS	194	53	0.06	0.3	8.48E-01	-0.06	0.3	8.54E-01	0.12	0.4	7.90E-01
Quinolinate	LC-MS	195	53	0.09	0.2	7.04E-01	0.18	0.2	4.35E-01	-0.09	0.3	7.78E-01
Tyramine	GC-MS	194	53	0.73	0.4	**4.72E-02**	0.33	0.4	3.71E-01	0.41	0.5	4.30E-01
3,4-dihydroxy-phenylalanine (DOPA)	GC-MS	194	53	0.53	0.6	3.74E-01	-1.10	0.6	5.96E-02	1.63	0.8	**4.96E-02**
Norepinephrine	GC-MS	194	53	0.89	0.5	5.28E-02	-0.09	0.5	8.36E-01	0.99	0.6	1.26E-01
**SERUM**												
**Aromatic amino acids and derivatives**												
Indolelactic acid	GC-MS	192	53	-0.21	0.1	7.51E-02	0.00	0.1	9.93E-01	-0.21	0.2	2.08E-01
Indoleacetic acid	LC-MS	194	53	0.00	0.1	9.42E-01	-0.05	0.1	4.07E-01	0.05	0.1	5.89E-01
Indolepropionic acid	LC-MS	194	53	0.02	0.0	6.02E-01	0.03	0.0	5.54E-01	0.00	0.1	9.59E-01
**Gut-brain**												
Kynurenine	LC-MS	194	53	0.01	0.1	9.41E-01	-0.04	0.1	7.06E-01	0.05	0.2	7.47E-01
Serotonin	LC-MS	194	53	-0.01	0.0	6.87E-01	0.02	0.0	4.05E-01	-0.03	0.0	3.78E-01
Glutamic acid	GC-MS	192	53	-0.16	0.1	2.37E-01	-0.09	0.1	5.15E-01	-0.07	0.2	7.11E-01
**Lipid metabolism**												
Linoleyl-carnitine	LC-MS	194	53	0.17	0.16	2.89E-01	-0.31	0.16	5.16E-02	0.47	0.22	**3.30E-02**
Octanoyl-carnitine	LC-MS	194	53	0.07	0.09	4.70E-01	-0.10	0.09	2.82E-01	0.16	0.13	2.00E-01
Linoleic acid	GC-MS	192	53	-0.18	0.14	2.17E-01	0.04	0.14	7.78E-01	-0.22	0.20	2.84E-01
Dodecanoyl-carnitine	LC-MS	194	53	0.11	0.11	2.96E-01	-0.03	0.11	7.90E-01	0.14	0.15	3.51E-01
Lauric acid	GC-MS	192	53	-0.16	0.14	2.38E-01	0.01	0.14	9.14E-01	-0.18	0.19	3.63E-01
β-aminoisobutyric acid	GC-MS	192	53	-0.26	0.23	2.55E-01	0.00	0.23	9.91E-01	-0.26	0.32	4.28E-01
Palmitoleic acid	GC-MS	192	53	-0.11	0.17	5.36E-01	0.06	0.17	7.30E-01	-0.17	0.24	4.95E-01
C4-carnitine	LC-MS	194	53	-0.02	0.05	6.46E-01	-0.07	0.05	1.59E-01	0.05	0.07	4.99E-01
Decanoyl-L-carnitine	LC-MS	194	53	0.05	0.10	5.71E-01	-0.04	0.10	7.07E-01	0.09	0.14	5.02E-01

1 Number of samples successfully analysed after removing individuals with only one sample, samples missing intestinal transit time measure and samples taken immediately after antibiotic treatment.

2 Number of successfully analysed after removing individuals with only one sample, samples missing intestinal transit time measure and samples taken immediately after antibiotic treatment.

The effect, standard error and P value of low-gluten vs. baseline and high-gluten vs. baseline are estimates of the mean change in the two periods derived from the LMM with an intervention-visit interaction adjusted for age, sex, intestinal transit time and with participant-specific and within-period participant-specific specific random effects. The effect, standard error and P value of low-gluten vs. high-gluten are estimates of differences in change (high-gluten relative to low-gluten) and derived from a similar LMM. Log transformation was applied to make data conform to normality. The list of metabolites was constructed from hypotheses based on results from the microbiome, urine metabolome and host response, hence no multiple test correction was applied.

Supplementary Table 13 Targeted associations between manually curated modules, BAIBA, kynurenine and serotonin

Variable 1	Data type variable 1	Variable 2	Data type variable 2	Spearman ρ	*P* value
Serotonin/Kynurenine production potential	Faecal metagenomics	Faecal Serotonin/Kynurenine	Faecal metabolite	0.20	4.08E-03
Serotonin/Kynurenine production potential	Faecal metagenomics	Serum Serotonin/Kynurenine	Serum metabolite	-0.04	5.50E-01
β-aminoisobutyric acid	Urine metabolite	Faecal Kynurenine	Faecal metabolite	0.26	9.23E-05
β-aminoisobutyric acid	Urine metabolite	Serum Kynurenine	Serum metabolite	-0.04	6.02E-01
β-aminoisobutyric acid	Urine metabolite	Faecal Serotonin	Faecal metabolite	0.07	2.89E-01
β-aminoisobutyric acid	Urine metabolite	Serum Serotonin	Serum metabolite	-0.02	8.02E-01
β-aminoisobutyric acid	Urine metabolite	Faecal Serotonin/Kynurenine	Faecal metabolite	-0.24	3.84E-04
β-aminoisobutyric acid	Urine metabolite	Serum Serotonin/Kynurenine	Serum metabolite	0.02	7.64E-01

The spearman coefficient and P value between two variables using a Spearman correlations with customized reference modules for serotonin and kynurenine production, faecal and serum kynurenine, faecal and serum serotonin and β-aminoisobutyric acid (BAIBA) as a continuous variables. Serotonin/Kynurenine production potential ((KYN008+KYN009)/KYN006). The modules were constructed to specifically target the significant metabolites and tested based on hypotheses, hence no multiple test correction was applied.

SUPPLEMENTARY FIGURES

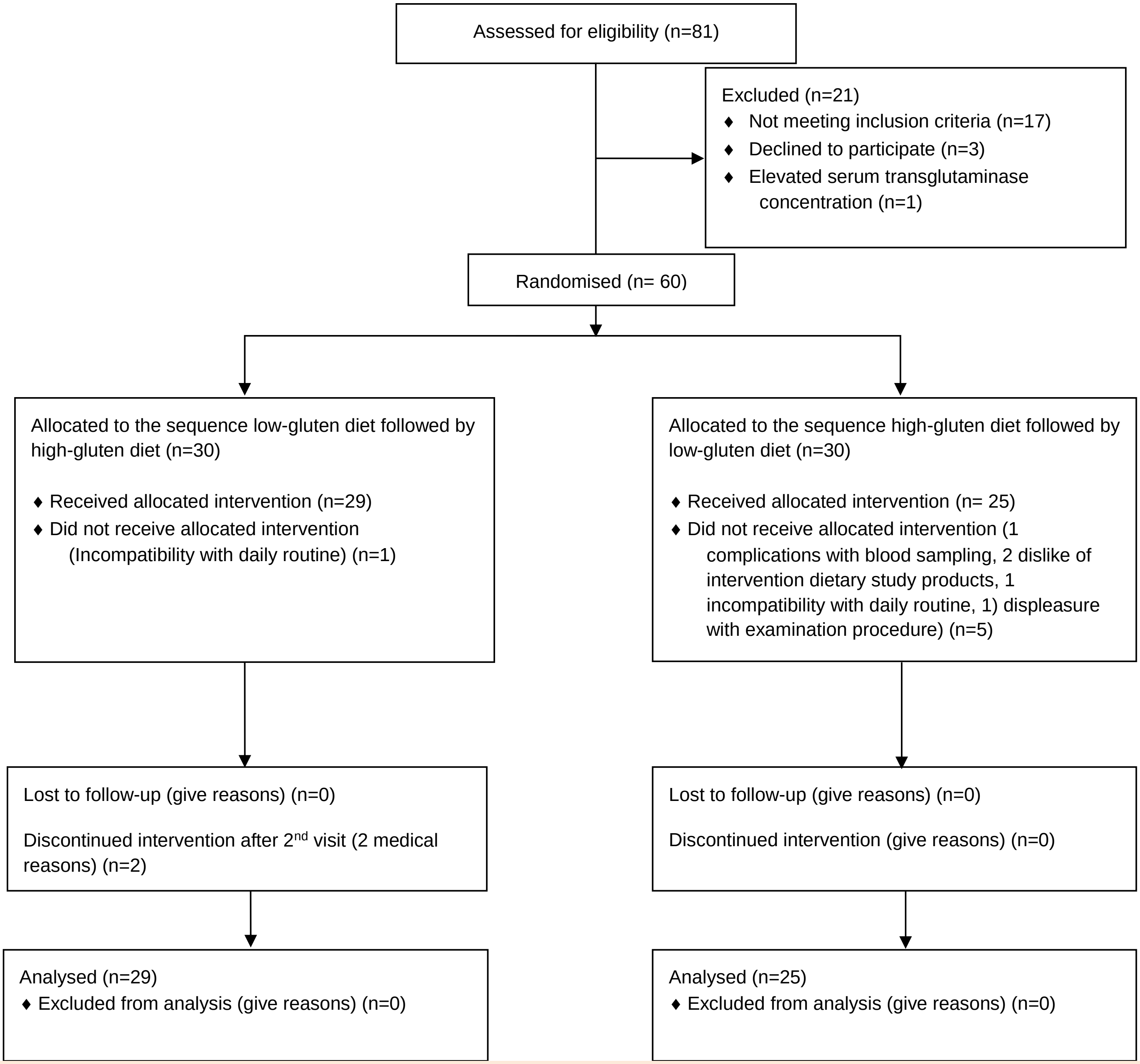

Statistical analysis of physiological measures: Samples excluded due to missing colonic transit time (CTT) data (samples n = 7) in statistical analyses, where physiological response was corrected for CTT.

Statistical analysis of the microbiome: Individuals excluded from the analysis based on low sequencing depth (n = 3). Individuals with incomplete dataset due to low sequencing depth (n = 2). Samples excluded due to missing CTT (samples n = 6) and samples excluded due to antibiotic treatment immediately before visit (samples n = 4). Total number of individuals included (n = 51). Total number of samples included (samples n = 188).

Supplementary Figure 1 CONSORT flow diagram for the study.

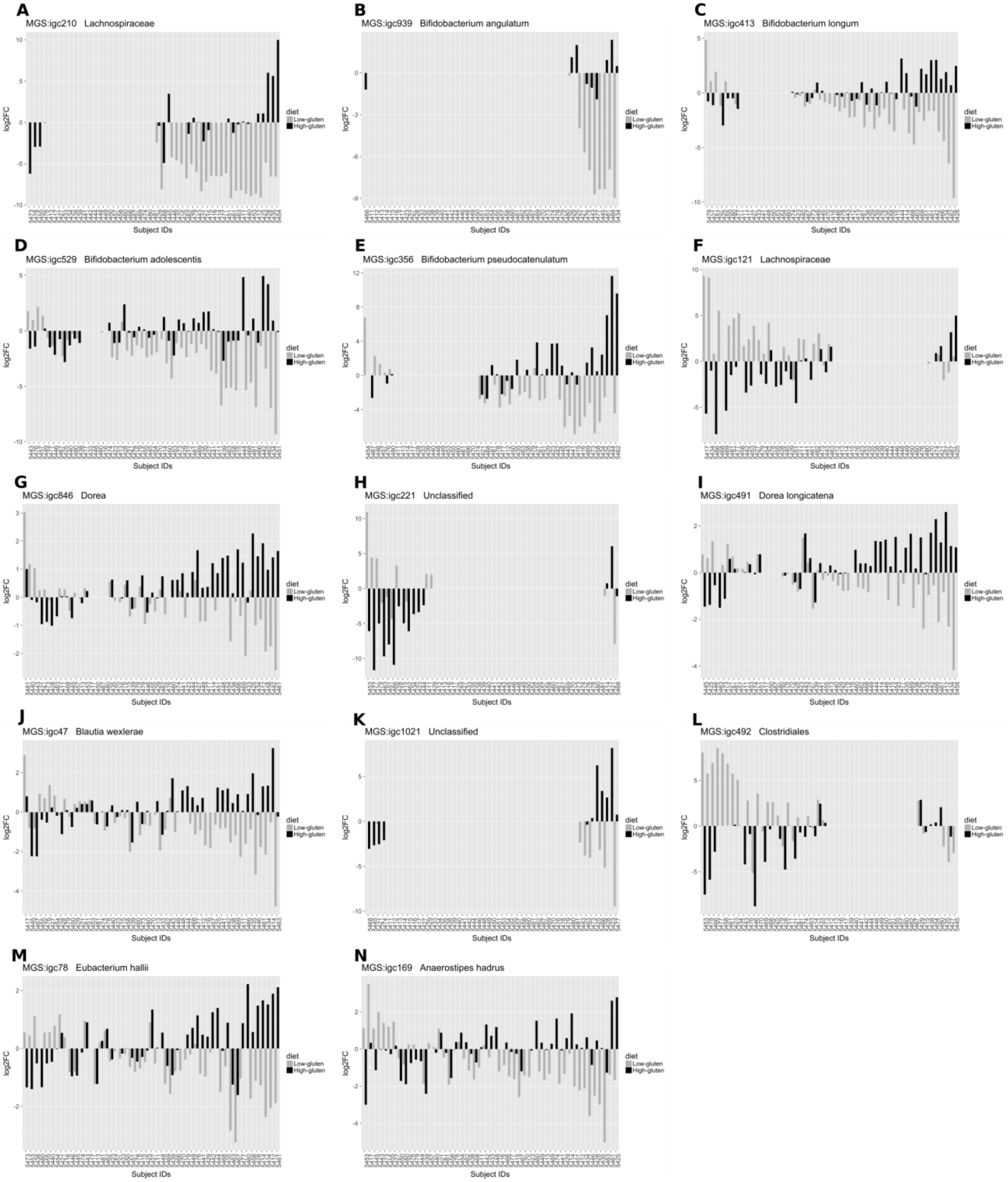

Supplementary Figure 2. Log2 fold change of abundance the significant MGSs between baseline and after the dietary intervention per subject.

The log2 fold change between baseline and after dietary intervention of abundance levels of the 14 significant MGSs illustrated for each subject. **a.** MGS:igc210 Lachnospiracaea. **b**. MGS:igc939 *B. angulatum*. **c.** MGS:igc413 *B. longum*. **d.** MGS:igc529 *B. adolescentis*. **e.** MGS:igc356 *B. pseudocatenulatum.* **f.** MGS:igc121 Lachospiraceae. **g.** MGS:igc846 *Dorea*. **h.** MGS:igc221 Unclassified. **i.** MGS:igc491 *Dorea longicatena*. **j.** MGS:igc47 *B. wexelera*. **k.** MGS:igc1021 Unclassified. **l.** MGS:igc492 Clostridiales. **m.** MGS:igc78 *E. halli*. **n.** MGS:igc169 *A. hadrus*.

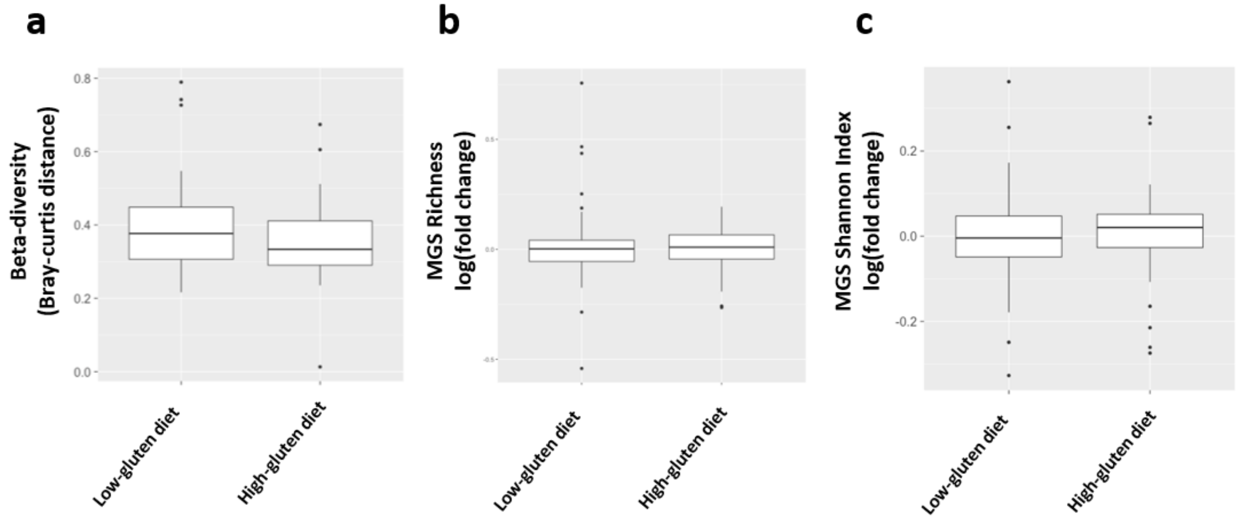

Supplementary Figure 3. Change in diversity of the gut microbiome during the interventions

Data are presented in a box-and-whiskers plot. **a**, No significant changes in beta-diversity as assessed by Bray-Curtis distances. **b-c**, alpha-diversity as assessed by fold change in metagenomic species (MGS) richness and MGS Shannon Index were observed comparing the low-gluten diet and the high-gluten diet. Changes in diversity was tested using linear mixed models adjusting for age, gender, intestinal transit time, participant (n = 51) and carry-over effect.

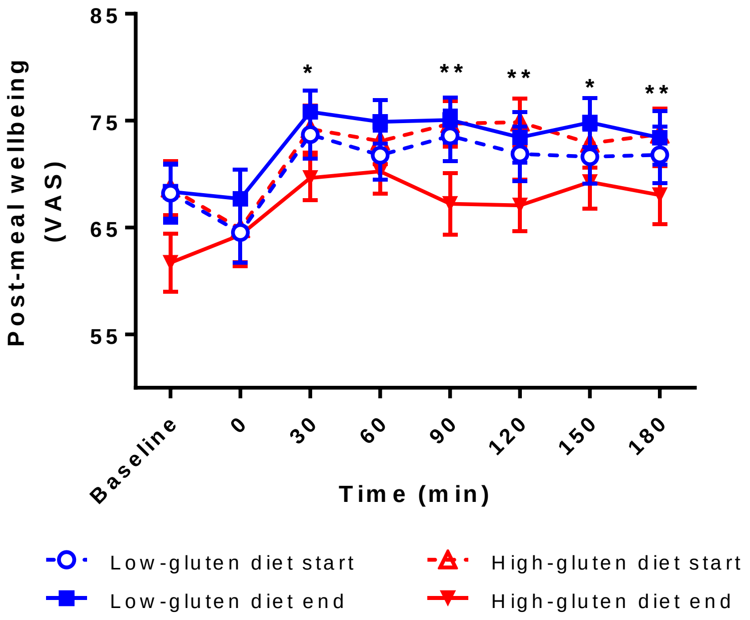

Supplementary Figure 4. Change in post-meal well-being following the dietary interventions

Post-meal wellbeing assessed by visual analogue scale (VAS) following the same standardised meal at all four visits. Data are shown as means±SEM (n=47-56). Changes were assessed by a linear mixed model adjusting for age, gender and intestinal transit time. * *P* <0.05, ** *P* < 0.01.

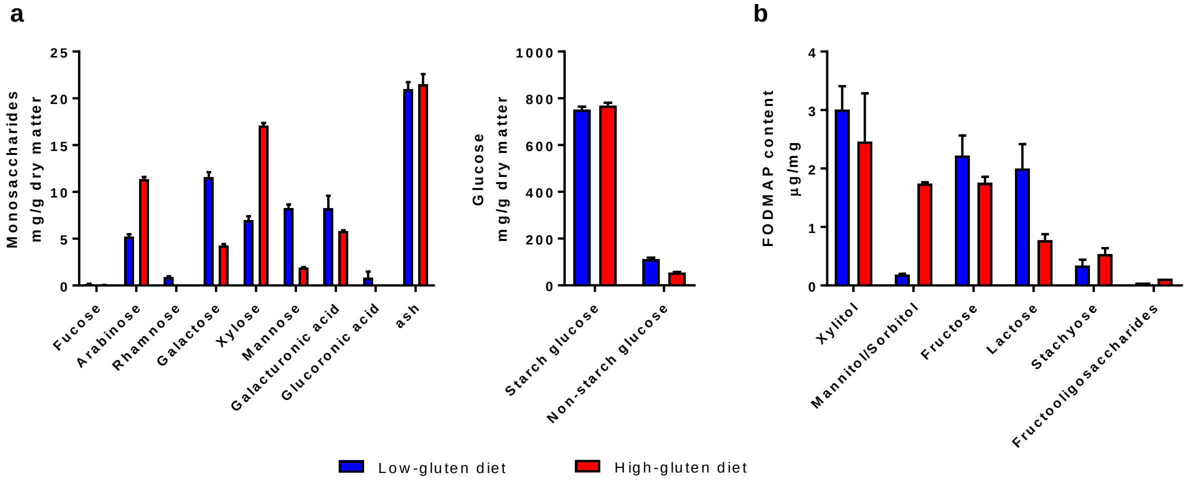

Supplementary Figure 5 Dietary fibre composition of the intervention diets.
a, Monosaccharides content of an average meal of the low-gluten diet and high-gluten diet based on daily intake from dietary records of the present study. **b**, FODMAP (Fermentable Oligosaccharides, Disaccharides, Monosaccharides and Polyols) composition of the provided low-gluten and the high-gluten study products**.** Data are mean ± SD (*n*=3).

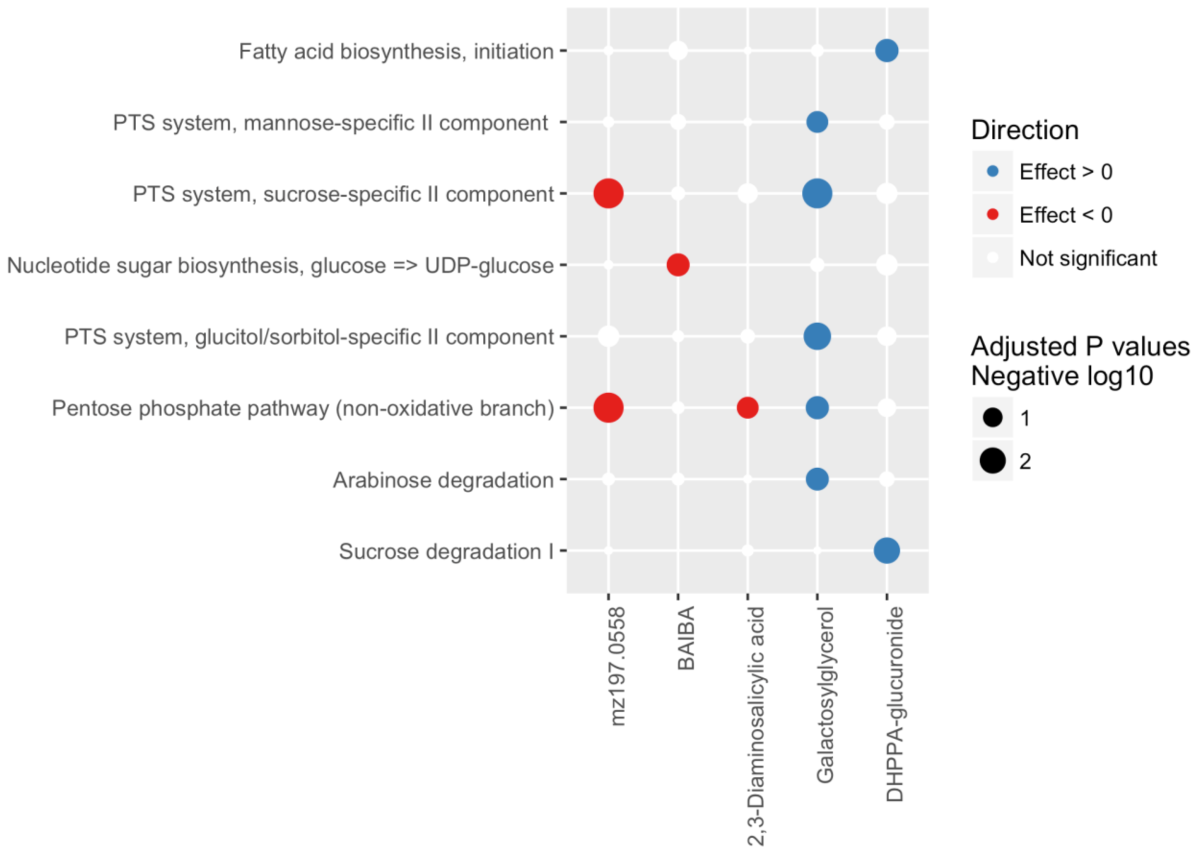

Supplementary Figure 6 Associations between significantly changing microbiome modules and urine metabolites. Linear mixed regression of modules and metabolites that are significantly changing due to the dietary intervention adjusted for age gender, intestinal transit time and participant. Only modules and metabolites with significant associations are included in the figure (FDR < 0.05), where positive (blue) and negative (red) associations are marked with coloured circles that are sized according to the negative log10 of the adjusted *P* value. *m/z*: mass-to charge

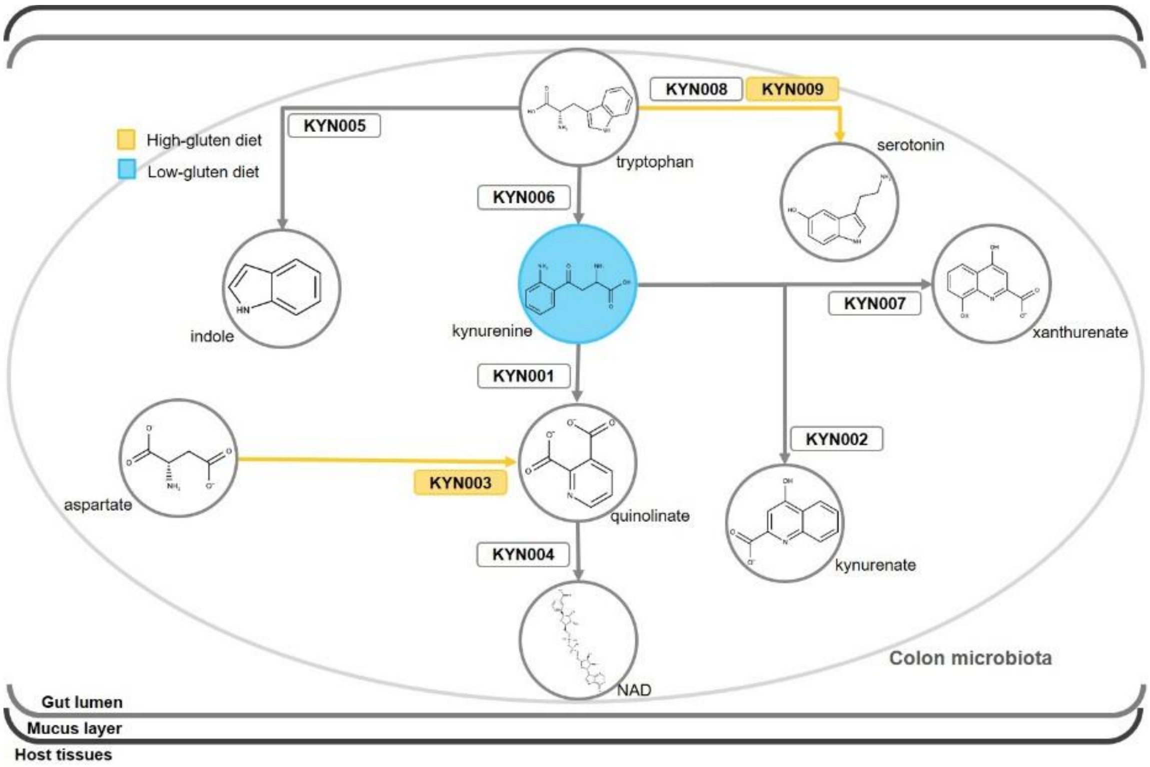

Supplementary Figure 7. Overview of kynurenine-related metabolic pathways as captured by the customized KYN module set (for pathway references, see **Supplementary Table 18**). Modules/metabolites with higher proportional abundances/faecal concentrations following the low-gluten diet are highlighted in blue, those positively associated to the high-gluten diet in yellow. Low-gluten dieting was associated with both higher faecal kynurenine concentrations and a lower proportional abundance of a serotonin production pathway. Together, both observations suggest a shift in faecal tryptophan metabolisation from serotonin (high-gluten diet) to kynurenine (low-gluten diet).

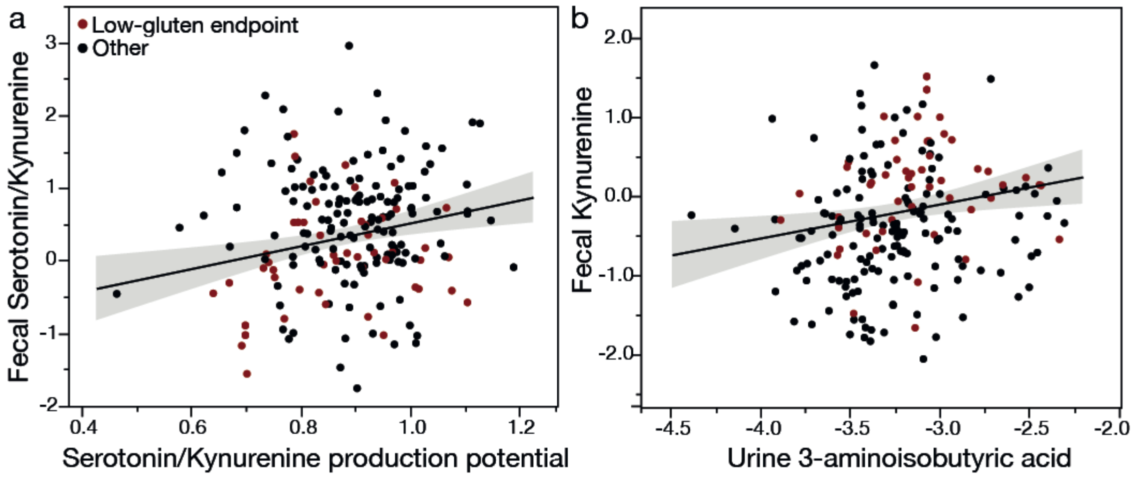

Supplementary Figure 8. Associations between tryptophan-derived metabolites serotonin and kynurenine faecal concentrations, the corresponding microbial metabolic production pathways, and urine 3-aminoisobutyric acid (BAIBA) levels. (a) Faecal Serotonin/Kynurenine metabolites ratio correlates to the respective faecal microbiota serotonin to kynurenine production potential ratio (Spearman rho=0.20; p=0.004, participants = 51, observations = 188). (b) Kynurenine faecal metabolite concentration correlates with urine BAIBA concentration (Spearman rho=0.26; p=9.2E-05, participants = 51, observations = 188).

SUPPLEMENTARY METHODS

Biochemical analyses of postprandial blood samples

Before plasma GLP-2 measurements, all samples were extracted in a final concentration of 75% ethanol, and at a final concentration of 70% ethanol before plasma GIP and plasma PYY measurements. Intact GLP-2 was measured using an in-house developed radio-immuno assay originally described elsewhere[5]. The antiserum (code no. 92160) is directed against the N-terminus of GLP-2 and therefore measures only fully processed GLP-2 of intestinal origin. For standards, we used recombinant human GLP-2 and the tracer was ^{125}I-labeled rat GLP-2 with an Asp33 -> Tyr33 substitution. Total GIP was measured using a C-terminally directed antiserum (code no. 80867), which reacts fully with intact GIP and N-terminally truncated forms as described elsewhere[6]. The standard was human GIP (Bachem, cat no. H-5645) and the tracer was ^{125}I-labeled human GIP (Perkin Elmer, cat no. Nex402). Total PYY was measured as described elsewhere[7] using a monoclonal antibody MAB8500 (Abnova, clone RPY-B12), which reacts equally well with $PYY_{1–36}$ and $PYY_{3–36}$. Synthetic human $PYY_{3–36}$ (Bachem, cat no. H-8585) was used as standard and ^{125}I-labeled PYY (Perkin Elmer, cat no. Nex341) as tracer. Sensitivity for all hormone assays was below 5 pmol L^{-1}, and intra-assay coefficient of variation below 10%. Plasma FFA was analysed using automated, enzymatic, colorimetric assay on ABX Pentra 400 chemistry analyzer (ABX Pentra, Horiba ABX, Montpellier, France). The CV% was 3.3%.

Dietary fibre composition of the two diets

A representative meal of each distinct diet was prepared based on daily intake from dietary records of the present study. The samples were freeze dried and milled to a ≤1 mm particle size. In order to determine the amount of starch, glucose and non-starch glucose, subsamples of the three diets were de-starched prior to hydrolysis. Samples were treated with 0.2% weight per volume Termamyl® SC for 85 min at 70°C in 50 mM phosphate buffer pH 6 according to Thomassen *et al.*[8]. Carbohydrate composition of the dietary fibre analysis was performed by a modified NREL sulphuric acid hydrolysis[9]. In brief, 150 mg sample was added to 1.5 mL 72% sulfuric acid, followed by incubation at 30°C for 60 min. Acid concentration was diluted to a final 4% sulfuric acid and the sample was autoclaved at 121°C for 60 min. The monosaccharide standards used for quantification were treated under the same conditions in order to take the recovery into account. Quantification was performed by High Pressure Anion-Exchange Chromatography system attached to Pulsed Amperometric Detection (HPAEC-PAD) BioLC system (Dionex, Sunnyvale, CA, USA) equipped with an AS50 autosampler, GS50 gradient pump and ED50 electrochemical detector equipped with a CarboPac PA1 analytical column (4x250 mm) and a guard column (4x50 mm) operated at 1 mL min^{-1} with isocratic elution in water for 38 min followed by isocratic elution in 500 mM NaOH for 15 min. Post-column addition of 500 mM NaOH at 0.2 mL min^{-1} was added for detection.

Resistant starch composition of the dietary study products

The percentage of digested starch in the dietary study products was evaluated by an *in vitro* static method that simulates physio-chemical conditions present in the human digestive system. Prior to the *in vitro* digestion processing, the food items were prepared according to instructions on the packet and snap-frozen in liquid nitrogen and stored before analysis. Before freezing, study

products cooked by boiling were drained of excess water. A standardised static *in vitro* digestion method[10] was used with slight modification. The simulated digestion fluids (Simulated Salivary Fluid (SSF) contained 15.1 mmol L^{-1} KCl, 13.6 mmol L^{-1} $NaHCO_3$, pH 7, Simulated Gastric Fluid (SGF) contained 6.9 mmol L^{-1} KCl, 47.2 mmol L^{-1} NaCl, 12.5 mmol L^{-1} $NaHCO_3$, pH 2) and Simulated Intestinal Fluid (SIF) contained 6.8 mmol L^{-1} KCl, 38.4 mmol L^{-1} NaCl, 85 mmol L^{-1} $NaHCO_3$, pH 7. These solutions were prepared to simulate the physio-chemical conditions present in the human body, but slightly simplified as compared to Minekus *et al.*[10]. For the oral phase the food was processed to simulate the chewing by using a mincer (Kitchen craft). Approximately 5 g (dry weight) of minced food substance (25°C) were mixed with SSF solution at the ratio of 1:1 (w:w) including $CaCl_2$ (0.75 mmol L^{-1}) and human salivary α-amylase (90 IU mL^{-1}, A1031 Sigma Aldrich). The bolus was incubated for 5 min at 37°C at 170 rpm using a Stuart orbital incubator SI500. For the gastric phase the oral bolus was mixed with one volume of SGF solution and $CaCl_2$ (0.075 mmol L^{-1}), porcine pepsin (1200 IU ml^{-1}) and fungal lipase (60 IU mL^{-1}) were added. Finally, for the intestinal phase the gastric chime was mixed with one volume of SIF stock solution and $CaCl_2$ (0.3 mmol L^{-1}) and pancreatic α-amylase (200 IU mL^{-1}) added using porcine pancreatin 8x, (P7545 Sigma Aldricht). The samples were incubated for 16 h at 37°C with 170 rpm mixing. Aliquots were withdrawn in triplicate for further analysis at different time-points to analyse the rapidly available sugars (RAS, oral and 30 min duodenal digestion) during the duodenal digestion, slowly digestible starch (SDS, starch digested between 30 and 120 min of duodenal digestion) and resistant starch (RS, 16 h). The digestion was stopped at the time points by adding one volume of 96% ethanol and increasing the pH to 10. Following the digestion, the samples were centrifuged at 10000 g for 10 min and the pellet was washed twice by using 80% ethanol. The supernatant and pellet were treated separately. Starch content in

the fractions was analysed using the AOAC method 996.11/AACC method 76.13. Glucose content was analysed by the PGO enzyme system (P7119 Sigma-Aldrich).

FODMAP composition of the dietary study products

Each dietary study product (10 g) was freeze-dried and finely grinded using a pestle and mortar. One g from each sample was pooled to generate one gluten-free and one non-gluten-free pool. Pooled sample (8 g) was suspended in 50 mL of distilled water, heated and stirred at 85°C for 25 min at 600 rpm. The extracts were spun down (1800 g, 1h). The supernatant was mixed with 4 volumes of 96% ethanol, incubated at 50°C for 10 min with mixing at 1000 rpm and pelleted at 10000g for 10 min. The supernatant containing FODMAPs was diluted 10 times with milliQ water and filtered through a 0.22 μm centrifuge filter. FODMAP analysis was performed using a HPAEC-PAD. Samples (15 μL) were injected and the separation was performed using a CarboPac™ SA10 column with a flow rate of 0.35 mL min^{-1}. The following gradient was applied: 0-10 mM NaOH linear gradient for 5 min, 10-500 mM concave gradient for 5 min, 500-800 mM convex gradient for 5 min, 800-50 mM linear gradient for 5 min, 50 mM isocratic for 15 min. Peaks were evaluated using Chromeleon V.6.80 and FODMAPs concentrations calculated by external authentic standards.

Metagenomic sequencing

DNA shearing and library preparations were performed according to the NEXTflex Rapid DNA-Seq Kit, V13.08 (Bioo Scientific, Austin, TX, USA). Briefly, 250 ng genomic DNA was sheared by Covaris E210 System using 10% duty cycle, intensity of 5, cycles per burst of 200 for 300 s to create 200 bp fragments. The samples were end-repair and adenylated to produce an A-overhang. Adapters containing unique barcodes were ligated on to the DNA. The samples were

then purified using the beads size selection for a selection range around 300-400 bp with the Agercount AMPure XP beads (Beckman Culter, Beverly, MA, USA). The purified DNA libraries were amplified with a denaturation time of 2 min at 98°C, followed by 12 cycles of denaturation at 98°C for 30 s, annealing at 65°C for 30 s and extension at 72°C for 1 min according to the protocol. The final extension was performed at 72°C for 4 min. Amplification was followed by DNA quantification using NanoDrop ND- 1000 UV-VIS Spectrophotometer (Thermo Fisher Scientific, Waltham, MA, USA) and the quality was checked on an Agilent 2100 Bioanalyzer using the Bioanalyser DNA High sensitivity (Agilent Technologies). Library preparation was performed by the DTU Multi-Assay Core (DMAC), Technical University of Denmark. The DNA libraries were mixed in equimolar ratios. Sequencing was performed as a 100 bp pair-end run on HiSeq 2000 (Illumina Int., San Diego, CA USA) at BGI Tech Europe (BGI Tech Europe, Copenhagen, Denmark) following the manufacturer's recommendations. The raw sequencing data for all 208 samples is available from the Short Read Archive under the Bioproject: PRJNA491335 [https://www.ncbi.nlm.nih.gov/bioproject/PRJNA491335].

Metagenomics analyses

The raw reads output was quality controlled using Cutadapt v. 1.8.1 with Python 2.7.10.[11] Bases with a Phred quality score below 20 were trimmed in both the 5' and 3' end of the sequences and reads shorter than 50 bases were discarded. Adaptor sequence remnants were removed in the 3' and 5' end of the reads. The total number of base pairs (bp) per sample ranged from 3.7 Gbp to 16.8 Gbp. Eight samples were discarded due to low sequence (< 3.7 Gbp) output. Trimmed reads were mapped against the Integrated Gene Catalogue (IGC)[12] comprising 9,879,896 genes derived from 1267 faecal samples, using BWA-MEM[13]. The read alignments were filtered using a 95% sequence similarity cut-off across the whole read and the number of reads mapping to a gene was

counted. Samples were rarefied using the R-package GUniFrac v 1.0 to the lowest sequence depth per individual to enable comparison within individuals, but not between individuals. This was done to ensure a minimum loss of information. For inter-individual analyses the gene abundances were rarefied to lowest sample sequence depth: 3.7 Gb.

The canopy clustering method described by Nielsen *et al*.[14] was applied on the 1267 samples from Li *et al*.[12] to create co-abundance gene clusters. Clusters containing more than 700 genes were assigned as metagenomic species (MGS) and amounted to 1264 MGSs. Abundances of genes belonging to such a cluster were used to calculate the overall relative abundance of a MGS. At least 5% of all genes belonging to a MGS should be observed before the MGS was detected; hence the 95th quantile of the abundances of the collection of genes from the same MGS was used as abundance measure. However, a minimum abundance cut-off was set to a 95th quantile of the read count to genes normalised by gene length (in bp) of less than 1e-4.

To use the full information contained within the genes of an MGS and to avoid potential missing marker genes, the taxonomy of the significant MGSs was determined by aligning all genes using Blast[15] against a collected database. The database was created by concatenating the following databases: Bacteria (23 Feb 2016), Archaea (24 Nov 2015), Fungi (24 Nov 2015), Non Flu Viruses (2 Sep 2015), Plasmid (23 Feb 2016), Protozoa (24 Nov 2015) and Virus (24 Nov 2015), which were downloaded as described by Nordahl *et al*.[16]

We only included Blast hits with an e-value lower than 1e-5 and an alignment length above 80% of the query length with an 80% sequence identity for determining the taxonomy. MGSs were classified as unknown if less than 15% of the genes had a Blast hit. MGSs were classified all the way to family level if more than 15% (min. 140) genes had a Blast hit and they consistently mapped to the same tax-id (minimum drop of 5% point). If more than 70% of the genes showed consistent

taxonomic annotation, the MGS was assigned to genus level and if more than 90% of the genes showed consistency, MGSs were mapped to species level.

The genes were annotated to the EggNOG database (v3) and the KEGG database as described by Li *et. al* [12]. Furthermore, manually curated modules were inferred from Vieira-Silva *et al.* 2016[17] and gene KEGG annotations. Given the significant association between dietary intervention and faecal concentrations of kynurenine, a specific metabolic reconstruction framework to interpret shotgun metagenomic data in terms of kynurenine metabolism potential was assembled based on extensive literature (**Supplementary Table 18**) and MetaCyc database[18]. A set of 9 modules (referred to as KYN) was assembled following the KEGG database syntax[19] as described in Vieira-Silva *et al*[17]. Each KYN module is delimited by its input and output compounds and encompasses all enzymes (ortholog groups) required to perform the reaction steps of all alternate pathways. When non-orthologous reactions for synthesis/utilization of a certain compound exist in prokaryotes, different KYNs were assembled for each. For each enzyme, the most precise prokaryotic-specific orthologous group containing all taxa that were experimentally proven to perform the function was selected, using the KEGG[19], TIGRFAM[20], and eggNOG[21] orthology databases in order of preference.

MGS and gene diversity were calculated by meant of the Shannon index and richness as number of MGS detected per rarefied sample (3.7 Gb). Beta diversity measures for MGS community composition and gene composition, respectively, were calculated using the Bray-Curtis measure (R-package ecodist v2.0.1[22]). The metagenomics data of 188 samples were used for statistical analyses after removing individuals with only one faecal sample (n=6), faecal samples with poor sequencing depth (n=4), faecal samples missing intestinal transit time measure (n=6) and faecal

samples affected by intake of antibiotics (n=4, phenoxymethylpenicillin, tetracyclin, ciprofloxacin or unknown antibiotics).

Quantitative PCR of *Bifidobacterium* spp. in faecal samples

The community DNA (stored at -80°C) extracted from 200mg ±20 mg faecal pellet (following centrifugation at 9500g for 5 min and removal of supernatant) from all faecal samples was diluted 100-fold prior to use as template in the quantitative PCR (qPCR) analysis. Quantification of *Bifidobacterium* spp. and total bacterial load was performed using genera specific primers (Forward primer: GCGTGCTTAACACATGCAAGTC and reverse primer: CACCCGTTTCCAGGAGCTATT)[23] and universal primers (HDA1: ACTCCTACGGGAGGCAGCAGT and HDA2: GTATTACCGCGGCTGCTGGCAC)[24] respectively, which target the bacterial 16S rRNA gene. To allow absolute quantification, standard curves were included of 10-fold dilution series of genomic DNA extracted from *Bifidobacterium longum* DSM 20219 and a linearized (SphI) plasmid standard, construction by cloning the 199bp 16S-V3 PCR amplification product of E. coli (ATCC 25922) into the pCR®4Blunt-TOPO vector (Invitrogen) as previously described[25]. The PCR reactions each contained 5.5 µl LightCycler® 480 II SYBR Green I Master (Roche Diagnostics A/S, Hvidovre, Denmark), 0.2µM of each primer and 2 µl template DNA in a total reaction volume of 11 µl. Reactions conditions were as follows: Initial 95ºC for 5 minutes, followed by 45 cycles of 95ºC for 10 seconds, 60ºC for 15 seconds and 72ºC for 45 seconds. Finally, a melting curve was generated by gradually increasing the temperature (95ºC for 5 seconds, 68ºC for 1 minutes and increasing the temperature to 98ºC with a rate of 0.11ºC per second with continuous fluorescence detection). The qPCR was performed in triplicate for each sample/primer combination in 384-well plate format on a LightCycler® 480 II instrument (Roche Applied Science) and analysed

using the dedicated LightCycler® 480 software for absolute quantification with standard curves. Samples were randomised between treatment groups and the four time-point, but all samples originating from a specific individual (4 samples x 3 replicates x 2 primer-sets = 24 samples) were always analysed on the same 384-well plate to reduce effects of plate-to-plate variation. Absolute abundances were calculated as bacterial genome equivalents per g faecal pellet and 16S gene copies per g faecal pellet for *Bifidobacterium* spp. and total bacterial load respectively. The relative abundance of *Bifidobacterium* spp. was calculated by dividing the calculated absolute abundance of *Bifidobacterium* spp. with the calculated total bacterial load.

Urine metabolomics by UPLC-MS

Acetonitrile and formic acid were obtained from Sigma-Aldrich (St. Louis, MO, USA). All aqueous solutions were prepared using ultrapure water obtained from a Millipore Milli-Q Gradient A10 system (Millipore, Bedford, MA). Enterolactone and 3,5-dihydroxyhydrocinnamic acid (DHPPA) were purchased from Sigma-Aldrich (Schnelldorf, Germany). β-aminoisobutyric acid (BAIBA) was purchased from Enamine Ltd (Kiev, Ukraine).

Exploratory non-targeted urinary metabolic profiling was performed by UPLC-MS with a quality control (QC) sample injected once for every 10 urine samples as previously reported[26]. The UPLC-MS system consisted of a Dionex Ultimate 3000 RS liquid chromatograph (Thermo Scientific) coupled to a Bruker maXis time-of-flight mass spectrometer equipped with an electrospray interphase (Bruker Daltonics). The analytes were separated on a Poroshell 120 SB-C18 column with dimensions of 2.1 × 100 mm and 2.7 µm particle size (Agilent Technologies). The column was held at 40 °C and the sampler at 4 °C. The UPLC mobile phases consisted of 0.1% formic acid in water (solution A) and 0.1% formic acid in acetonitrile (solution B). With a constant flow rate of 0.4 ml min–1, the analytes were eluted using the following gradient.

Solvent programming was isocratic 1% B for 1 min followed by a linear gradient up to 15% at 3 min, then a linear gradient up to 50% B at 6 min and finally a linear gradient up to 95% B at 9 min. The final gradient composition, 95% B, was held constant until 10 min, followed by a return of the solvent composition to initial conditions at 10.1 min and re-equilibration until 13 min. Mass spectrometry data were collected in full scan mode at 2 Hz with a scan range of 50–1,000 mass/charge (m/z). The following electrospray interphase settings were used: nebulizer pressure 2 bar, drying gas 10 l min–1, 200 °C, capillary voltage 4,500 V. For tandem mass spectrometry (MS/MS) analyses, a ramp collision energy ranging from 10 to 30 eV was applied on a scheduled precursor list. To improve the measurement accuracy, external and internal calibrations were performed using sodium formate clusters (Sigma-Aldrich), and a lock-mass calibration was also applied (hexakis(1H,1H,2H-perfluoroetoxy) phosphazene, Apollo Scientific). The raw UPLC-MS data were converted to mzXML files using Bruker Compass DataAnalysis 4.2 software (Bruker Daltonics) and were then pre-processed through noise filtering, peak detection and alignment using the open-source R package XCMS (v.1.38.0)65. Noise filtering settings included that features should be detected in a minimum of 80% of samples. Data tables were generated comprising m/z, retention time and intensity (peak area) for each variable in every sample. The data were normalized to the total intensity. Subsequently, the data were filtered using the pooled QC samples; features with coefficient of variation above 0.3 in the QC samples were excluded, and features with a retention time below 0.5 min or above 8 min were excluded. The CV% of the metabolites in the QC samples measured by UPLC-MS in positive (1299 features) and negative (232 features) ionisation mode was on average 8% and 13%, respectively. The accurate masses of the discriminating features measured by UPLC-MS were searched for putative molecules in the METLIN[27] and HMDB[28] databases. For the identification of UPLC-MS metabolite candidates, discriminating features were subjected to

MS/MS experiments. The metabolites were identified according to the four different levels described by the Metabolomics Standard Initiative;[3] metabolites confirmed by an authentic standard (Level I), metabolites confirmed based on a comparison of MS/MS fragmentation pattern compared with those found in databases and earlier literature (Level II), metabolites with similarities to published fragmentation patterns (Level III), and unknown compounds (Level IV). To confirm the identity of sulfonated and glucuronidated metabolites, deconjugation experiments using β-glucuronidase from *E. coli* K12 (Roche Diagnostics GmbH) and sulfatase from *Aerobacter aerogenes* (Sigma-Aldrich, Schnelldorf, Germany) were performed. For each deconjugation reaction, urine samples were diluted in sodium phosphate buffer (pH=7.4), incubated with the enzymes at 37°C for 1-2 h, injected onto the UPLC-MS for analysis, and compared to standards when available.

Urine metabolomics by GC-MS

All chemicals used for urine metabolic profiling by GC-MS in the study were analytical grade. Sodium hydroxide, pyridine, methyl chloroformate (MCF), anhydrous sodium sulfate, sodium bicarbonate, the internal standard 2,3,3,3-d_4-DL-alanine and the external C7-C30 saturated alkanes standard were obtained from Sigma-Aldrich (St. Louis, MO, USA). Chloroform, methanol and hexane were obtained from Merck (Darmstadt, Germany). The GC-MS analyses were performed as previously described[11]. Briefly, urine samples were derivatised by MCF and injected on a GC-MS (Agilent 7890A coupled to MSD 5975C) with a mass selective detector (EI) operating at 70 eV. The column used for all analyses was a TG-1701MS GC column (30m x 0.255mm x 0.1µm with 5m guard column, Thermo Fisher, USA). Data processing was done in MSD ChemStation and AMDIS software (NIST, Boulder, CO, USA). The GC-MS data (peak height) were normalised by the internal standard 2,3,3,3-d_4-DL-alanine and the measured urine

creatinine concentration to account for the technical variability associated with chemical derivatisation and the dilution of urine, respectively. Identification of metabolites was done using our in-house MCF MS library, which contains information of mass fragmentation and retention times of more than 300 authentic standards and by use of the commercially available library from National Institute of Standards and Technology (NIST).

Serum and faeces metabolomics

All serum and faeces samples were randomised prior to extraction and analysis. Extraction of metabolites from serum was carried out essentially as previously described[29]. Briefly, four volumes of ice-cold methanol supplemented with ^{13}C- and ^{15}N-labeled amino acids (6 μM, MSK-A2-1.2, Cambridge Stable Isotopes) and 2-ethyl butyrate (5 μM) as internal standards was added to serum, vortexed and incubated on ice for 15 minutes. Samples were centrifuged and supernatants transferred to a new tube. For faeces metabolomics four volumes of ice-cold 50% methanol supplemented with ^{13}C- and 15N-labeled amino acids (6 μM) and 2-ethyl butyrate (100 μM) as internal standards were added to faecal samples, vortexed and incubated on ice for 15 minutes. Samples were centrifuged and supernatants were filtered through a PTFE 0.2 μm spin filter (Thermo). Prior to lyophilisation an aliquot of the supernatant was transferred to a new tube, and used for analysis of short-chain fatty acids.

GC-MS Analysis of extracted metabolites from serum and faeces was carried out as previously described[29]. After lyophilization metabolites were subjected to methoxyamine/MSFTA derivatization and subsequently analysed by GC-MS. Samples were analysed on an Agilent 7200 GC/Q-TOF equipped with a DB-5MS-UI column (Agilent). Data inspection, data mining,

annotation and interpretation were done in MassHunter, Profinder and MPP (all Agilent Technologies, Santa Clara, CA, USA).

Metabolites in serum and faeces extracts were profiled using LC-MS. Lyophilized samples were redissolved in 1% formic acid (FA) in water before injection on a Agilent 1290 Ininity HPLC system (Agilent Technologies, Santa Clara, CA) equipped with an Agilent Zorbax Eclipse Plus C18 column (2.1 x 150 mm, 1.8 μm) with a 50 mm guard column, both kept at 40°C. The chromatographic gradient was run at a flow rate of 300 μL min^{-1} with the following solvent composition of A (0.1 % FA, water) and B (0.1% FA, acetonitrile): 97% A from 0-5 min, 97-85% A from 5-8 min and 85-60% A from 8-18 min before equilibration for 3 min with the initial conditions. Samples were analysed twice with the MS operating in both positive and negative ion mode, respectively. One master mix sample (equal amounts pooled from thirty random samples) was included for all setups and run in all-ion fragmentation mode with collision energy of 20 V in order to produce fragments for identification of the metabolites. The LC flow was coupled to an Agilent 6530 quadrupole time of flight (Q-TOF) mass spectrometer scanning from 70-1050 m/z. Libraries of metabolites with retention time were constructed using Agilent MassHunter PCDL Manager. The identification of each compound was based on exact mass, retention time of synthetic standards and/or comparison of fragments with the Metlin MS/MS database (https://metlin.scripps.edu/). Chromatograms for all compounds were extracted and quantified using Agilent Profinder using a mass tolerance of 20 ppm and a retention time tolerance of 0.1 min.

SCFA in plasma and faeces by LC-MS

Analysis of short-chain fatty acids in serum and faecal extracts were carried out as previously described[30], except that derivatives were separated on a on a Agilent 1290 Ininity HPLC system

(Agilent Technologies, Santa Clara, CA) equipped with an Agilent Zorbax Eclipse Plus C18 column (2.1 x 150 mm, 1.8 μm) with a 50 mm guard column, both kept at 40°C and identified on a Agilent 6530 quadrupole time of flight (Q-TOF) mass spectrometer.

Statistical analyses

For testing the host physiology, all factors described above were included in the LMM, except when testing intestinal transit time, where transit time as a factor was excluded. An interaction term of intervention and visit was included in the LMM with seven levels including all combinations of intervention (2 levels) and visit (4 levels: visit 1=baseline 1, visit 2, visit 3=post-randomization baseline 2, visit 4). The baseline at visit 1 was assumed to be the same for the two randomized groups, hence visit 1 was combined to one level. This design allowed for carry-over effects since baseline at visit 3 preceded a specific intervention. The intervention effect was estimated directly by a *post-hoc* t-test as the average differences of the differences between baseline and intervention, hence between visits 1 and 2 and between visits 3 and 4. The effects of the low-gluten intervention and high-gluten intervention compared to the habitual diets at baseline were also tested.

All physiological measures were assessed for linearity using histograms, residual plot and QQ-plot of the residuals. TAG, ALAT, ASAT, CRP, IL-6, TNF-α, HOMA-IR, insulin, haemolysis and exhalation of hydrogen were all log-transformed to accommodate linearity. The visual analogue scoring of personal gastrointestinal indicators were zero inflated due to the nature of the questions, why the scorings were dichotomized, where scores below 5 mm were set to zero. The gastrointestinal indicators were tested using binominal distribution and responders were subsequently tested using the LMM.

For the postprandial analyses of blood biochemistry, exhalation of hydrogen and gut wellbeing questionnaire with multiple time-points for one visit, a three-way interaction-term of visit-intervention and time after the standardized meal was tested. Assuming that measures closer in time would correlate more, we implemented a Gaussian covariance structure, which assumes measurements of the same participant to be serially correlated within one visit. For each time point, the intervention effect was evaluated and a χ^2-test was used.

For the cytokine measures after *ex vivo* LPS stimulation, triplicates were averaged and tested using the LMM as previously described but including mix of monocytes, eosinophils, basophils and mast cells (MXD) as a factor for TNF-α, IL-6 and IL-1β and IFN-γ was corrected for lymphocytes (LYM) .

Change in plasma levels of alkylresorcinols were analysed using a LMM adjusted for changes in intestinal transit time, serum triglycerides and low-density lipoprotein-cholesterol concentration and participant as a random variable[2]. For comparison of end-points of the two periods, a paired t-test or a Wilcoxon matched pairs test was used depending on whether the data were normally distributed.

All 989 out of a total of 1264 species present in at least 2 individuals were tested using the LMM and adjusted for participant, participant x period term, age, gender and intestinal transit time as described above. The participant was included in the analysis if the MGS was observed in at least one of the four time points. In cases, where all participants included were of the same gender, this factor was excluded from the model. All abundances were naturally log-transformed after adding the lowest abundance to all numbers to avoid infinity measures. Similar to the host physiology analyses, an intervention-visit term was tested. Due to the natural individual variance in the gut microbiome composition, eight levels of the intervention-visit term were included to

allow for differences at baseline between the two randomised groups besides including individuals as a factor to allow for a unique intersect at baseline. *P* values were corrected for multiple testing using the Benjamini-Hochberg false discovery rate (FDR)[4].

Histograms, residual plot and QQ-plot of residuals were used to assess the compliance of normality of the significant MGSs. Significant MGSs with zero inflation were confirmed using a mixed model with binary distribution followed by a linear mixed model test of all non-zero values.

Concentrations of *Bifidobacterium* (*B. longum* DSM 20219 genomes per g faeces) calculated with standard curve, total bacterial load, *Bifidobacterium* relative abundance calculated with standard curve and *Bifidobacterium* relative abundance calculated with Ct method asses by qPCR were all log transformed to conform normality and evaluated for intervention effects using the linear mixed model.

All 6739 KOs present in more than two study participants were tested using a LMM as described above with an eight-level intervention-visit interaction-term. All KOs present in less than 10 individuals were excluded and the smallest value was added to all abundances before natural log-tranformation of the data. LMMs were applied to test the modules including a three-way term of intervention-visit-KO, where the effect on each KO was tested and globally summarized using a χ^2-test. The average effect size, *P* values and adjusted *P* values were reported. To evaluate the contribution of the significant MGSs to the significance of the modules, a similar analysis was conducted where genes from the significant MGSs were excluded from the dataset and adjusted *P* values were reported.

Changes in urine metabolites were analysed using the described LMM with eight levels of the

intervention-visit term. We adjusted *P* values using the Benjamini-Hochberg FDR[4].

To evaluate intervention effects on the targeted metabolites measured in serum and faeces, all data were log transformed to conform normality and tested using the described LMM with eight levels of the intervention-visit term.

To test the association between the significant outcomes of MGSs, exhalation of hydrogen and urine metabolites a pairwise regression analysis was conducted. The LMMs included the outcomes as continuous variables, participant as a random effect and age and gender as fixed effects.

The association between significant modules, hydrogen exhalation and urine metabolites were also evaluated using LMM adjusted for participant as a random effect and age, gender and intestinal transit time as fixed effects. Specifically, LMMs were fitted for each KO within a significant module and slope coefficients for the association of interest were extracted from all model fits and used obtaining a pooled slope coefficient that was interpreted as the module-level measure of the association. *P* values were adjusted for multiple comparisons using the Benjamini-Hochberg FDR[4].

The code used to compute abundances of the kynurenine specific modules (KYN) from a KO abundance table is shared on GitHub (https://github.com/raeslab/GMMs), where KYN abundances were computed as the median orthologue group abundance of the maximum alternative pathway. Renormalization by using ratios between sub-components of the matrix is a way to circumvent the compositionality of metagenomic data[31]. We thus used the ratio between microbial serotonin production potential (KYN008, KYN009) and kynurenine production potential (KYN006). We

correlated this ratio (Serotonin/Kynurenine production potential) to the respective ratio in metabolite concentrations in faecal and serum samples.

SUPPLEMENTARY REFERENCES

1. Saxholt, E. *et al.* Danish Food Composition Databank, Revision 7. *Department of Nutrition, National Food Institute, Technical University of Denmark* (2008).

2. Lind, M. V *et al.* Plasma Alkylresorcinols Reflect Gluten Intake and Distinguish between Gluten-Rich and Gluten-Poor Diets in a Population at Risk of Metabolic Syndrome. *J. Nutr.* **146,** 1991–1998 (2016).

3. Sumner, L. W. *et al.* Proposed minimum reporting standards for chemical analysis Chemical Analysis Working Group (CAWG) Metabolomics Standards Initiative (MSI). *Metabolomics* **3,** 211–221 (2007).

4. Benjamini, Y. & Hochberg, Y. Controlling the False Discovery Rate - a Practical and Powerful Approach to Multiple Testing. *J. R. Stat. Soc. Ser. B-Methodological* **57,** 289–300 (1995).

5. Hartmann, B. *et al.* Structure, measurement, and secretion of human glucagon-like peptide-2. *Peptides* **21,** 73–80 (2000).

6. Lindgren, O. *et al.* Incretin hormone and insulin responses to oral versus intravenous lipid administration in humans. *J Clin Endocrinol Metab* **96,** 2519–2524 (2011).

7. Torang, S. *et al.* In vivo and in vitro degradation of peptide YY3-36 to inactive peptide YY3-34 in humans. *Am J Physiol Regul Integr Comp Physiol* **310,** R866-74 (2016).

8. Thomassen, L. V & Meyer, A. S. Statistically designed optimisation of enzyme catalysed starch removal from potato pulp.

9. Sluiter, A. *et al.* Determination of structural carbohydrates and lignin in biomass. *Lab. Anal. Proced.* **1617,** (2008).

10. Minekus, M. *et al.* A standardised static in vitro digestion method suitable for food–an international consensus. *Food Funct.* **5,** 1113–1124 (2014).

11. Roager, H. M. *et al.* Whole grain-rich diet reduces body weight and systemic low-grade inflammation without inducing major changes of the gut microbiome: a randomised cross-over trial. *Gut* gutjnl-2017-314786 (2017). doi:10.1136/gutjnl-2017-314786

12. Li, J. *et al.* An integrated catalog of reference genes in the human gut microbiome. *Nat. Biotechnol.* **32,** 834–841 (2014).

13. Li, H. & Durbin, R. Fast and accurate long-read alignment with Burrows-Wheeler transform. *Bioinformatics* **26,** 589–95 (2010).

14. Nielsen, H. B. *et al.* Identification and assembly of genomes and genetic elements in complex metagenomic samples without using reference genomes. *Nat. Biotechnol.* **32,** 822–8 (2014).

15. Altschul, S. F., Gish, W., Miller, W., Myers, E. W. & Lipman, D. J. Basic local alignment

search tool. *J. Mol. Biol.* **215,** 403–410 (1990).

16. Nordahl Petersen, T. *et al.* Meta-genomic analysis of toilet waste from long distance flights; a step towards global surveillance of infectious diseases and antimicrobial resistance. *Sci. Rep.* **5,** 11444 (2015).

17. Vieira-Silva, S. *et al.* Species–function relationships shape ecological properties of the human gut microbiome. *Nat. Microbiol.* **1,** 16088 (2016).

18. Caspi, R. *et al.* The MetaCyc database of metabolic pathways and enzymes and the BioCyc collection of Pathway/Genome Databases. *Nucleic Acids Res.* **42,** D459-71 (2014).

19. Kanehisa, M. *et al.* Data, information, knowledge and principle: back to metabolism in KEGG. *Nucleic Acids Res.* **42,** D199–D205 (2014).

20. Haft, D. H. *et al.* TIGRFAMs and Genome Properties in 2013. *Nucleic Acids Res.* **41,** D387-95 (2013).

21. Powell, S. *et al.* eggNOG v3.0: orthologous groups covering 1133 organisms at 41 different taxonomic ranges. *Nucleic Acids Res.* **40,** D284-9 (2012).

22. Goslee, S. C. & Urban, D. L. The ecodist Package for Dissimilarity-based Analysis of Ecological Data. *JSS J. Stat. Softw.* **22,** (2007).

23. Penders, J. *et al.* Quantification of *Bifidobacterium* spp., *Escherichia coli* and *Clostridium difficile* in faecal samples of breast-fed and formula-fed infants by real-time PCR. *FEMS Microbiol. Lett.* **243,** 141–147 (2005).

24. Walter, J. *et al.* Detection and identification of gastrointestinal Lactobacillus species by using denaturing gradient gel electrophoresis and species-specific PCR primers. *Appl. Environ. Microbiol.* **66,** 297–303 (2000).

25. Tulstrup, M. V.-L. *et al.* Antibiotic Treatment Affects Intestinal Permeability and Gut Microbial Composition in Wistar Rats Dependent on Antibiotic Class. *PLoS One* **10,** e0144854 (2015).

26. Roager, H. M. *et al.* Colonic transit time is related to bacterial metabolism and mucosal turnover in the gut. *Nat. Microbiol.* **1,** 16093 (2016).

27. Smith, C. *et al.* METLIN: a metabolite mass spectral database. *Ther. Drug Monit.* **27,** 747–751 (2005).

28. Wishart, D. S. *et al.* HMDB 3.0--The Human Metabolome Database in 2013. *Nucleic Acids Res.* **41,** D801–D807 (2013).

29. Berg, S. M. *et al.* The heterozygous N291S mutation in the lipoprotein lipase gene impairs whole-body insulin sensitivity and affects a distinct set of plasma metabolites in humans. *J. Clin. Lipidol.* **11,** 515–523.e6 (2017).

30. Han, J., Lin, K., Sequeira, C. & Borchers, C. H. An isotope-labeled chemical

derivatization method for the quantitation of short-chain fatty acids in human feces by liquid chromatography-tandem mass spectrometry. *Anal. Chim. Acta* **854,** 86–94 (2015).

31. Gloor, G. B., Macklaim, J. M., Pawlowsky-Glahn, V. & Egozcue, J. J. Microbiome Datasets Are Compositional: And This Is Not Optional. *Front. Microbiol.* **8,** 2224 (2017).
