# Supplementary material for: A low-gluten diet induces changes in the intestinal microbiome of healthy Danish adults
Source: Nat Commun. 2018 Nov 13;9:4630. doi: 10.1038/s41467-018-07019-x (PMC6234216; doi:10.1038/s41467-018-07019-x)
Supplement: Supplementary file 2 — Description of Additional Supplementary Files [file 41467_2018_7019_MOESM2_ESM.pdf]

Description of Additional Supplementary Files

File Name: Supplementary Data 1

Description: Metagenomics sequencing results for each stool sample. Data are rarefied intra-individually (using data from the four visits) and presented as raw reads, trimmed reads, raw base pairs, trimmed base pairs and reads mapping to the integrated gene catalog.

File Name: Supplementary Data 2

Description: Metagenomic species (MGSs) changing during the low-gluten and high-gluten interventions. The effect, standard error and P value of low-gluten vs. baseline and high-gluten vs. baseline are estimates of the mean change in the two periods derived from the LMM with an intervention-visit interaction adjusted for age, sex, intestinal transit time and with participant-specific and within-period participant-specific specific random effects. The effect, standard error and P value of low-gluten vs. high-gluten are estimates of differences in change (high-gluten relative to low-gluten) and derived from a similar LMM.

File Name: Supplementary Data 3

Description: Difference in change in KOs during the low-gluten and high-gluten interventions. The effect, standard error and P value of low-gluten vs. baseline and high-gluten vs. baseline are estimates of the mean change in the two periods derived from the LMM with an intervention-visit interaction adjusted for age, sex, intestinal transit time and with participant-specific and within-period participant-specific specific random effects. The effect, standard error and P value of low-gluten vs. high-gluten are estimates of differences in change (high-gluten relative to low-gluten) and derived from a similar LMM.

File Name: Supplementary Data 4

Description: Difference in change in KEGGs and manually curated (customised) reference modules during the low-gluten and high-gluten interventions. The effect, standard error and P value of low-gluten vs. baseline and high-gluten vs. baseline are estimates of the mean change in the two periods derived from the LMM with an intervention-visit interaction adjusted for age, sex, intestinal transit time and with participant-specific and within-period participant-specific specific random effects. The effect, standard error and P value of low-gluten vs. high-gluten are estimates of differences in change (high-gluten relative to low-gluten) and derived from a similar LMM. Individual KOs in the modules were tested using LMM against the intervention and then summarized using a chi square test. GBM: gut-brain interaction modules, GIM: gut microbiota immune-interaction modules, GMM: gut-specific metabolic modules.

File Name: Supplementary Data 5

Description: Associations between differing significant MGS, urine metabolites and breath hydrogen. The effect, standard error and P value are estimates of the correlations between mean changes in two variables using a LMM with MGS as response and urine metabolites and breath hydrogen as a continuous variable when correcting for age, gender and individual as a random factor. m/z: mass-to-charge.

File Name: Supplementary Data 6

Description: Composition and statistics of customised kynurenine modules. The structure of the customised modules describing the kynurenine-related metabolic pathways from the eggNOG, KEGG and Tigr databases including pathway references and the average effect and P value of low-gluten vs. baseline and high-gluten vs. baseline are estimates of the mean change in the two periods derived from the LMM with an intervention-visit interaction adjusted for age, sex, intestinal transit time and with participant (n=51) and within-period participant-specific specific random effects. The average effect and P value of low-gluten vs. high-gluten are estimates of differences in change (high-gluten relative to low-gluten) and derived from a similar LMM. Individual KOs in the modules were tested using LMM against the intervention and then summarized using a chi square test.
