# Supplementary material for: A low-gluten diet induces changes in the intestinal microbiome of healthy Danish adults
Source: Nat Commun. 2018 Nov 13;9:4630. doi: 10.1038/s41467-018-07019-x (PMC6234216; doi:10.1038/s41467-018-07019-x)
Supplement: Supplementary file 9 — Reporting Summary [file 41467_2018_7019_MOESM9_ESM.pdf]

nature research

Corresponding author(s): Oluf Pedersen, NCOMMS-17-32523A-Z

# Reporting Summary

Nature Research wishes to improve the reproducibility of the work that we publish. This form provides structure for consistency and transparency in reporting. For further information on Nature Research policies, see Authors & Referees and the Editorial Policy Checklist.

## Statistical parameters

When statistical analyses are reported, confirm that the following items are present in the relevant location (e.g. figure legend, table legend, main text, or Methods section).

| n/a | Confirmed | |
|---|---|---|
| ☐ | ☒ | The exact sample size ($n$) for each experimental group/condition, given as a discrete number and unit of measurement |
| ☐ | ☒ | An indication of whether measurements were taken from distinct samples or whether the same sample was measured repeatedly |
| ☐ | ☒ | The statistical test(s) used AND whether they are one- or two-sided<br>*Only common tests should be described solely by name; describe more complex techniques in the Methods section.* |
| ☐ | ☒ | A description of all covariates tested |
| ☐ | ☒ | A description of any assumptions or corrections, such as tests of normality and adjustment for multiple comparisons |
| ☐ | ☒ | A full description of the statistics including central tendency (e.g. means) or other basic estimates (e.g. regression coefficient) AND variation (e.g. standard deviation) or associated estimates of uncertainty (e.g. confidence intervals) |
| ☐ | ☒ | For null hypothesis testing, the test statistic (e.g. $F$, $t$, $r$) with confidence intervals, effect sizes, degrees of freedom and $P$ value noted<br>*Give P values as exact values whenever suitable.* |
| ☒ | ☐ | For Bayesian analysis, information on the choice of priors and Markov chain Monte Carlo settings |
| ☒ | ☐ | For hierarchical and complex designs, identification of the appropriate level for tests and full reporting of outcomes |
| ☐ | ☒ | Estimates of effect sizes (e.g. Cohen's $d$, Pearson's $r$), indicating how they were calculated |
| ☐ | ☒ | Clearly defined error bars<br>*State explicitly what error bars represent (e.g. SD, SE, CI)* |

*Our web collection on statistics for biologists may be useful.*

## Software and code

Policy information about availability of computer code

| | |
|---|---|
| Data collection | No software was used |
| Data analysis | All statistical analyses were performed in R version 3.1 (The R Foundation for Statistical Computing, 2012, Vienna, Austria). |

For manuscripts utilizing custom algorithms or software that are central to the research but not yet described in published literature, software must be made available to editors/reviewers upon request. We strongly encourage code deposition in a community repository (e.g. GitHub). See the Nature Research guidelines for submitting code & software for further information.

## Data

Policy information about availability of data

All manuscripts must include a data availability statement. This statement should provide the following information, where applicable:
- Accession codes, unique identifiers, or web links for publicly available datasets
- A list of figures that have associated raw data
- A description of any restrictions on data availability

The raw Illumina read data for all samples are available from the Short Read Archive under the Bioproject: PRJNA491335 [https://www.ncbi.nlm.nih.gov/bioproject/PRJNA491335].

# Field-specific reporting

Please select the best fit for your research. If you are not sure, read the appropriate sections before making your selection.

☒ Life sciences ☐ Behavioural & social sciences ☐ Ecological, evolutionary & environmental sciences

For a reference copy of the document with all sections, see nature.com/authors/policies/ReportingSummary-flat.pdf

# Life sciences study design

All studies must disclose on these points even when the disclosure is negative.

| | |
|---|---|
| Sample size | Estimations were based on 85 % statistical power to detect a difference of 0.4 standard deviation in metabolic quantitative traits, based on previous observations from the MetaHit study (Le Chatelier et al, 2013; Nature). It was estimated that 51 individuals were needed, but to allow for a 15 % drop-out after randomization, a total of 60 participants were invited for participation.<br>Additionally, based on observed standard deviations for the metagenomic species (MGSs) changing during the gluten-poor and gluten-rich interventions, we concluded that the number of included subjects was adequate to provide evidence of a changed intestinal microbiome after a gluten-poor diet compared with a gluten-rich diet. |
| Data exclusions | Data from four individuals at visit 2 or 3 were excluded due to use of antibiotics. |
| Replication | N/A due to the nature of an dietary intervention. |
| Randomization | 60 participants were randomized at the first examination day. Randomization was performed separately for each of the studies in blocks of variable size to ensure equal randomization throughout the enrolment phase of the study. The randomization sequence was made by an investigator without contact<br>to the participants (www.randomization.com). |
| Blinding | Both, the participants and the investigators involved in outcome assessment were blinded until the first examination day. Thereafter, blinding was not feasible due to<br>the nature of the intervention. However, blinding of the allocation sequence was re-established during sample analysis and initial data analysis. |

# Reporting for specific materials, systems and methods

## Materials & experimental systems

| n/a | Involved in the study | |
|---|---|---|
| ☒ | ☐ | Unique biological materials |
| ☐ | ☒ | Antibodies |
| ☒ | ☐ | Eukaryotic cell lines |
| ☒ | ☐ | Palaeontology |
| ☒ | ☐ | Animals and other organisms |
| ☐ | ☒ | Human research participants |

## Methods

| n/a | Involved in the study | |
|---|---|---|
| ☒ | ☐ | ChIP-seq |
| ☒ | ☐ | Flow cytometry |
| ☒ | ☐ | MRI-based neuroimaging |

## Antibodies

| | |
|---|---|
| Antibodies used | Total GIP was measured using a C-terminally directed antiserum (code no. 80867) and total PYY was measured using a monoclonal antibody MAB8500 (clone RPY-B12; Abnova, Taipei, Taiwan) |
| Validation | Validation is described in Lindgren, O. et al. Incretin hormone and insulin responses to oral versus intravenous lipid administration in humans. J Clin Endocrinol Metab 96, 2519–2524 (2011).<br>Torang, S. et al. In vivo and in vitro degradation of peptide YY3-36 to inactive peptide YY3-34 in humans. Am J Physiol Regul Integr Comp Physiol 310, R866-74 (2016). |

## Human research participants

Policy information about studies involving human research participants

| | |
|---|---|
| Population characteristics | Sixty Caucasian Danish adults without known chronic disorder including gastrointestinal disease, coeliac disease or diabetes. |

| | |
|---|---|
| Population characteristics | They were between 22 and 65 years old, apparently healthy, weight stable and had a body mass index (BMI) of 25-35 kg/m2 and/or increased waist circumference (≥94 cm for men and ≥80 cm for women). |
| Recruitment | Participants were recruited from the general population studies “Health 2008” and “Health 2010”, established at the Research Center for Prevention and Health (RCPH) at Glostrup University Hospital in Copenhagen, Denmark and through the webpage www.forsogsperson.dk and advertisements in local newspapers. |
